# Development of adjustable variable stiffness restrainer for bridge subjected to seismic excitation

**DOI:** 10.1371/journal.pone.0286977

**Published:** 2023-06-15

**Authors:** Mustafa Kareem Hamzah, Farzad Hejazi

**Affiliations:** 1 Department of Civil Engineering, University of Warith Al-Anbiyaa, Karbala, Iraq; 2 Faculty of Environment and Technology, The University of The West England, Bristol, United Kingdom; Tongji University, CHINA

## Abstract

This paper presents a numerical and experimental assessment of a developed adjustable variable stiffness restrainer (AVSR) utilized for short span bridges. This restrainer has the ability to demonstrate multi stiffness capacity in different stages of bridge’s superstructure movement to mitigate the severe damage of bridge due to an earthquake. The multi-level stiffness behavior of developed AVSR is achieved by using multiple mechanical springs with different lengths and placed in parallel in proposed design. A small prototype of developed AVSR has been fabricated and tested under incremental and cyclic loading in order to assess the restrainer performance and the behavior has been validated using finite element analysis. Thereafter, the constitutive model of AVSR was derived for the proposed restrainer in order to implement it in numerical simulations. Furthermore, a parametric study has been conducted numerically to evaluate the effectiveness of different parameters on the restrainer capacity. Moreover, the efficiency of AVSR application in a single degree of freedom system has been assessed by performing seismic analysis on a frame equipped with AVSR subjected to different seismic excitations using Newmarkʼs method. The experimental and finite element results proved the efficiency of developed variable stiffness device to exhibit adjustable action against imposed loads in three designed stages. Furthermore, the parametric study results revealed that increasing the section area of the spring wire leads to increase the restrainer capacity. In contrast, the restrainer resistance is declined by an increase in the mean spring diameter and number of coils for each spring of AVSR. The time history analysis results also indicated that the frame response in terms of displacement, velocity and acceleration is improved by implementing the AVSR in the considered system.

## Introduction

One of the most essential concerns of structural engineers is bridge resistance toward dynamic loading such as wind, earthquake and etc [1]. The severe damage consequences to bridges in the previous Chi-Chi [2, 3], Kobe [4] and Kocaeli seismic excitations [5] showed that it is required to develop new methods in order to diminish the severe impact of seismic excitations on bridges. Implementation of restrainers or dampers in bridges for improving lateral resistance, ductility and unseating prevention without scarifying the overall integrity of bridges is one of the most effective techniques that can guarantee the structural safety of bridges. Recently, different researches have been conducted to examine the impact of implementing different types of damping systems in the bridge to control the bridge movement under applied loads [6, 7]. Numerical simulations and experimental testing have widely been performed on restrainers, dampers and bridges equipped with these devices [8–10]. The results revealed that utilizing the bridge with supplementary damping system is resulting to improve the bridges performance during seismic excitation [11–13].

Variable stiffness systems are widely implemented in the structures due to their capability to minimize impact of severe ground motion. The principle of variable stiffness systems is to gradually increase the stiffness of the structure or bridge [14]. There are numerous advantages to utilize the adjustable variable stiffness systems since in the low stiffness, the flexible structure is able to bear small deformations due to daily applied loads, however, the higher stiffness of the system is leaded to protect the structures against applied excessive movements [15]. Therefore, the high stiffness will improve the structure’s overall seismic performance and prevent of any serious damages [16]. Thus, different variable stiffness devices were developed by numerous researchers. The constitutive models were derived based on elastic theory and the seismic performance of Single Degree of Freedom frame equipped with different types of variable stiffness devices has been assessed using Newmark’s method. The results showed that the variable stiffness devices were efficient in reducing the impact of different ground motions [17–19]. Furthermore, a spring type restrainer was developed to mitigate the seismic effect on curved bridges [20]. The bridge’s seismic performance has been assessed numerically with the proposed restrainer and subjected to three earthquakes. The results revealed that the developed device was able to diminish the severe ground motion impact. In (2019), a pre-compressed spring in X-cable was used for the braced frame [21]. The researchers conducted experimental testing and numerical analysis to evaluate the cyclic performance of the frame and they concluded that the cable reached its ultimate capacity along with the compressed spring without losing the cable. As a consequence, the variable stiffness property will control the seismic effect on bridges.

Self-centering capability is one of the most notable advantages of damping or restrainer systems that considered by recent studies. The shape memory alloy (SMA) is a smart material that is widely used to demonstrate the self-centering capability. Thus, recently different restrainers systems have been developed using shape memory alloy material. Liang, D., et al. (2020) developed a cable controlled sliding bearing SMA and compared it with a traditional steel restrainer. The hybrid SMA system showed considerable resistance, comprehensive self-centering capability in addition to supplementary damping during severe ground motion. Furthermore, the steel cable showed a pier damage and should be replaced after an earthquake, in contrast with the SMA system, which demonstrated less damage and did not require replacement after ground motion [22]. Cao, S., et al. (2020) proposed a hybrid multi-level bearing system (SMA cable and conventional lead rubber bearing) that has the ability to increase the resistance with earthquake intensity incrementally. Three different groups of SMA-cables with different characteristics were utilized to achieve the desired multi-level behavior. The bearing protection system exhibited a valuable resistance during different levels of ground motion. Furthermore, the system demonstrated the ability to reduce excessive bearing movement [23]. Fang, C., et al. (2021, 2022) performed comprehensive studies about utilizing SMA cables to mitigate the severe impact of earthquakes. A new combination between SMA cables and high damping rubber isolator has been introduced. The outcomes explained the importance of equipping the structures with SMA technologies to diminish the earthquake effect [24, 25]. However, the disadvantage of using smart material such as SMA beside their high cost is behavior and performance of these materials, which is highly affected by temperature.

For the most of active and semi-active variable stiffness systems, an external power source is required to operate the device, which may lead to decline the device functionality during sever excitations. Furthermore, these devices are required for frequent repairs or replacements of some parts after experiencing of earthquake. Although the active and semi-active variable stiffness systems have complex mechanisms or a high cost, which make them difficult to manufacture and conduct experimental investigations. As a result, it is necessary to propose a new system that does not rely on a power source or is affected by temperature and requires routine maintenance.

Therefore, this study presents a new design for a variable stiffness restrainer with a simple structure, cost effective and does not depend on external power for its functioning. Then, the mathematical and analytical models for stiffness of proposed AVSR have been formulated for various stages of imposed loads (movement). Thereafter, the functioning of device has been evaluated experimentally through conducting incremental load and cyclic test and also numerically through assessing the performance of AVSR in a single degree of freedom system subjected to earthquake excitation.

## Adjustable Variable Stiffness Restrainer (AVSR)

**Fig 1:**
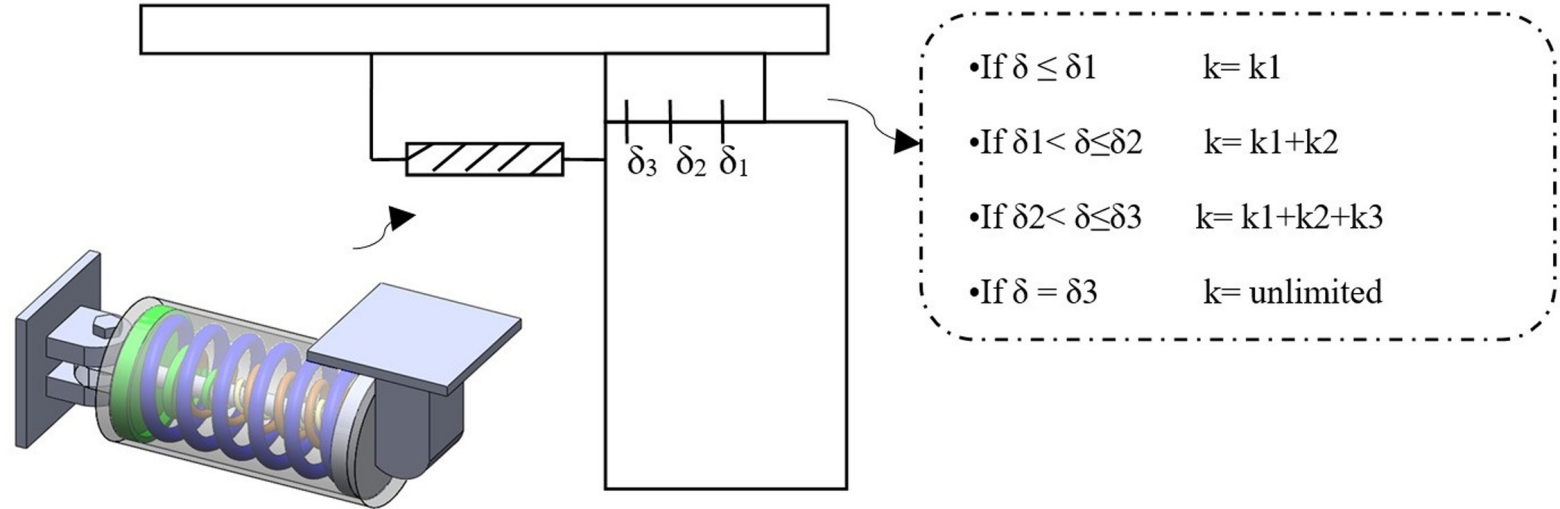
Adjustable Variable Stiffness Restrainer (AVSR).

The main components of the proposed AVSR are three or more helix-coil springs with different characteristics. The placement of springs is arranged through position of an inner spring inside two others in parallel in order to achieve the parallel functioning of springs and obtain the variable stiffness behavior of the restrainer as depicted in Fig 1. Hence, the bridge girder movement can be controlled at various stages of movement through utilizing of AVSR. Thus, the AVSR in low stiffness action can shift the bridge period away from the earthquake period and in high stiffness function, it is able to prevent the bridge failure due to excessive movement. A steel shaft is positioned at the center of springs as their core and in order to control the spring’s movement, a steel cover has been attached to both ends of each spring. Then, springs are tied to the support from one side through welding and end covers align the movement of the spring from another side along with the steel shaft that is located in the center of springs.

The arrangement of springs is designed to implement the contribution of each spring in the 3 sequence ranges of restrainer deformation. Therefore, at the first range of movement (beginning of movement) only one spring with lower stiffness is functioned and by increasing the movement to pass through second and third deformation ranges, second and third springs which have a higher stiffness are functioned and contribute to the action of AVSR. After compression of all springs to their limits, the restrainer will function as a rigid stopper and prevent the bridge’s girder from failing. The restrainer parameters such as spring’s characteristics, materials, and number of springs are designed based on required stiffness to restrain the movement of bridge deck. The proposed restrainer can be installed between an abutment and girder or bent to girder of bridges through a bolted or welded connection. As mentioned before, throughout the proposed design for AVSR, when the ground motion is applied to the bridge and then through bridge deck to the restrainer, only the outer spring is functional in the first operation range of displacement(δ1) by providing a low stiffness of k1. By increasing the movement due to the earthquake, the displacement is increased to the ranges within δ1 and δ2, and then the stiffness of the AVSR is increased to k2 by involving the second spring with the restrainer functionality until cretin displacement (second limit). Then, the restrainer stiffness is highly increased and reaches k3 by involving the inner spring with the other two springs when the displacement is between ranges of δ2 and δ3. Finally, when the displacement reaches the last mist of δ3, the AVSR functions as a rigid stopper since all the springs are compressed to their limited function.

## Mathematical formulations for AVSR

The main component of AVSR is the spring; in this study, two types of springs have been selected (normal coiled spring and die spring), since the die spring will be selected in heavy-duty applications. For this type of spring the stiffness equation will be:

$$\text{Range}\;\text{(i):}\;\text{If}\;\delta \;\leq \;\delta_{1},\;k\;=\;k1\;\text{So},\;k\;=\;\frac{Gd1^{4}}{8D1^{3}n1}$$ (1)

$$\text{Range}\;\text{(ii):}\;\text{If}\;\delta_{1}\;<\;\delta \;\leq \;\delta_{2},\;k\;=\;k1\;+\;k2\;\text{So},\;k\;=\;\frac{D_{2}^{3}n_{2}Gd_{1}^{4}+D_{1}^{3}n_{1}Gd_{2}^{4}}{8D_{1}^{3}D_{2}^{3}n_{2}n_{1}}$$ (2)

$$\text{Range}\;\text{(iii):}\;\text{If}\;\delta_{2}\;<\;\delta \;\leq \;\delta_{3},\;k\;=\;k1\;+\;k2\;+\;k3\;\text{So},\;k\;=\;\frac{D_{2}^{3}D_{3}^{3}n_{2}n_{3}Gd_{1}^{4}+D_{1}^{3}D_{3}^{3}n_{1}n_{3}Gd_{2}^{4}+D_{1}^{3}D_{2}^{3}n_{1}n_{2}Gd_{3}^{4}}{8D_{1}^{3}D_{2}^{3}D_{3}^{3}n_{1}n_{2}n_{3}}$$ (3)

Where: d = wire diameter, D = mean diameter, n = number of coils, G = Shear modulus. Eqs. 1, 2, and 3 represent the variable stiffness behavior of the circular wire device.

For the rectangular wire (die) spring, a partial deferential equation should be satisfied for spring deflections [26] as:

$$\frac{\partial^{2}z}{\partial x^{2}}\;+\;\frac{\partial^{2}z}{\partial y^{2}}\;=\;\;-\;\frac{q}{s}$$ (4)

**Fig 2:**
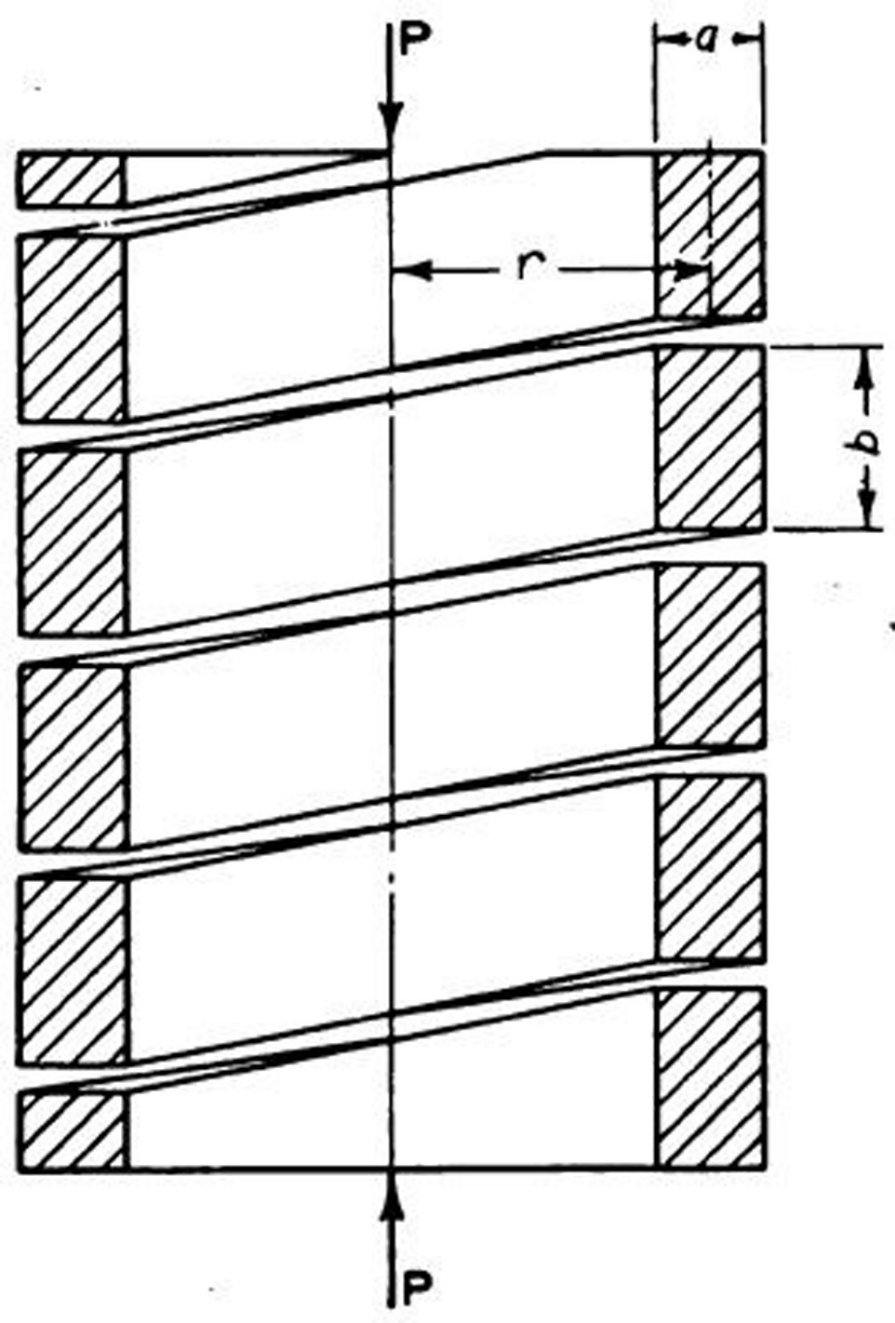
Axially loaded rectangular spring [26].

Where z represents the membrane deflection at a point with x and y coordinates. While q and S represent the pressure on area of the membrane and the tension force in the membrane itself per unit length respectively. Moreover, z should equal zero at the edges. Fig 2 demonstrates the rectangular cross section with dimensions a and b, in addition to the fact that b is bigger than a. Therefore, the deflection will be written in series form as [26]:

$$Z\;=\;\sum_{n=1,3,5,\ldots }^{\infty }b_{n}\cos\frac{n\pi x}{a}\cdot Yn$$ (5)

Where, Y represents the function of y only. Eq (4) will be satisfied by a suitable choice of Y. The expression for Y will be determined by substitution of Eq (5) in Eq (4) and expanding Eq (5) right side in the form of Fourier’s series, as follows:

$$z\;=\;\frac{4qa^{2}}{S\pi^{3}}\;*\;\sum_{n=1,3,5,\ldots }^{\infty }\frac{1}{n^{3}}\;(-1)\;\frac{n-1}{2}\;\left(1\;-\;\frac{\cosh\frac{n\pi y}{a}}{\cosh\frac{n\pi b}{2a}}\right)\cos\frac{n\pi x}{a}$$ (6)

The shear stress at any point is related to the slope of the membrane at the corresponding point. Therefore, the q/S has been replaced by 2*Gθ*, *G* represents the shear modulus, and *θ* is the angular twisting per unit length. The maximum shear (τ_m_) can be achieved by differentiating Eq (6); y will be taken as 0 and x equal to a/2 as follows:

$$\tau_{m}\;=\;\frac{8G\theta a}{\pi^{2}}\;*\;\sum_{n=1,3,5,\ldots .}^{\infty }\frac{1}{n^{2}}\left(1\;-\;\frac{1}{\cosh\frac{n\pi b}{2a}}\right)$$ (7)

By considering double the volume (*v*) under the deflection membrane, the torsion (M) will be determined in terms of angular twisting and q/S will again be replaced by 2*Gθ*.


$$v\;=\;2\int_{-\frac{a}{2}}^{+\frac{a}{2}}\int_{-\frac{b}{2}}^{+\frac{b}{2}}zdxdy$$ (8)

$$M\;=\;G\theta a^{3}b\left(\frac{1}{3}\;-\;0.209\;\;*\;\;\frac{a}{b}\left(\tanh\frac{\pi b}{2a}\;+\;0.004\right)\right)$$ (9)

For simplicity, this equation may be written:

$$M\;=\;¥G\theta a^{3}b$$ (10)

Where ¥ is equal to:

$$¥\;=\;\left(\frac{1}{3}\;-\;0.209\;*\;\frac{a}{b}\left(\tanh\frac{\pi b}{2a}\;+\;0.004\right)\right)$$ (11)

Then, solve for *θ*:

$$\theta \;=\;\frac{M}{¥a^{3}bG}$$ (12)

For a rectangular-wire helical spring, the twisting moment:

M=Fr (13)

Where, r is the mean radius of the spring

Since *θ* can be calculated from Eq (12) and the torque is equal to the applied force times the spring mean radius. Therefore, the deflection will be:

$$\delta \;=\;2\pi nr^{2}\theta$$ (14)

By implementing the value of *θ*, the deflection will be:

$$\delta \;=\;\frac{2\pi Fr^{3}n}{Ga^{3}\left(\frac{b}{3}\;-\;0.209a\left(\tanh\frac{\pi b}{2a}\;+\;0.004\right)\right)}$$ (15)

By implementing Eq (11) in Eq (15), the spring deflection will be:

$$\delta \;=\;\frac{2\pi Fr^{3}n}{¥a^{3}bG}$$ (16)

Flexibility can be achieved by derivation of x (f):

$$\frac{dx}{df}\;=\;\frac{2\pi r^{3}n}{¥a^{3}bG}$$ (17)

Stiffness is the inverse of flexibility:

$$k\;=\;\frac{¥a^{3}bG}{2\pi r^{3}n}$$ (18)

Range (i): If δ ≤ δ_1_, k = k_1_

$$k\;=\;\frac{{¥}_{1}a_{1}^{3}b_{1}G}{2\pi r_{1}^{3}n_{1}}$$ (19)

Range (ii): If δ_1_< δ ≤ δ_2_, k = k_1_+k_2_

$$k\;=\;\frac{{¥}_{1}a_{1}^{3}b_{1}G}{2\pi r_{1}^{3}n_{1}}\;+\;\frac{{¥}_{2}a_{2}^{3}b_{2}G}{2\pi r_{2}^{3}n_{2}}$$

$$k\;=\;\frac{r_{2}^{3}n_{2}{¥}_{1}a_{1}^{3}b_{1}G\;+\;r_{1}^{3}n_{1}{¥}_{2}a_{2}^{3}b_{2}G}{2\pi r_{1}^{3}r_{2}^{3}n_{1}n_{2}}$$ (20)

Range (iii): If δ_2_< δ ≤ δ_3_, k = k_1_+k_2_+k_3_

$$k\;=\;\frac{r_{2}^{3}n_{2}{¥}_{1}a_{1}^{3}b_{1}G+r_{1}^{3}n_{1}{\left(k_{1}\right)}_{2}a_{2}^{3}b_{2}G}{2\pi r_{1}^{3}r_{2}^{3}n_{1}n_{2}}\;+\;\frac{{¥}_{3}a_{3}^{3}b_{3}G}{2\pi r_{3}^{3}n_{3}}$$

$$k\;=\;\frac{r_{3}^{3}n_{3}\left(r_{2}^{3}n_{2}{¥}_{1}a_{1}^{3}b_{1}G+r_{1}^{3}n_{1}{¥}_{2}a_{2}^{3}b_{2}G\right)\;\;+\;\;r_{1}^{3}r_{2}^{3}n_{1}n_{2}{¥}_{3}a_{3}^{3}b_{3}G}{2\pi r_{1}^{3}r_{2}^{3}r_{3}^{3}n_{1}n_{2}n_{3}}$$

$$k\;=\;\frac{r_{2}^{3}r_{3}^{3}n_{2}n_{3}{¥}_{1}a_{1}^{3}b_{1}G\;+\;r_{1}^{3}r_{3}^{3}n_{1}n_{3}{¥}_{2}a_{2}^{3}b_{2}G\;+\;r_{1}^{3}r_{2}^{3}n_{1}n_{2}{¥}_{3}a_{3}^{3}b_{3}G}{2\pi r_{1}^{3}r_{2}^{3}r_{3}^{3}n_{1}n_{2}n_{3}}$$ (21)

Eqs (19), (20) and (21) represent the variable stiffness behavior of the rectangular wire device.

The following steps show the simplified design process of the proposed AVSR:

1. Define the bridge properties and requirement based on code of practice.
2. Perform static and dynamic analysis using finite element program.
3. Determine the seat length and girder dead load reaction via analysis results.
4. Determine the required restrainer capacity and overall displacement based on the considered code of practice.
5. Determine the displacement limit for the three levels based on the constitutive model.
6. Perform the nonlinear analysis to check the bridge responses such as pounding potential or unseating of bridge girder for evaluation of the code criteria.
7. If the bridge response is satisfied the code conditions, then selected properties can be considered as final details of AVSR.
8. Otherwise, reselect restrainer specifications to obtain the acceptable bridge response.

## Numerical and experimental evaluation of AVSR prototype

**Fig 3:**
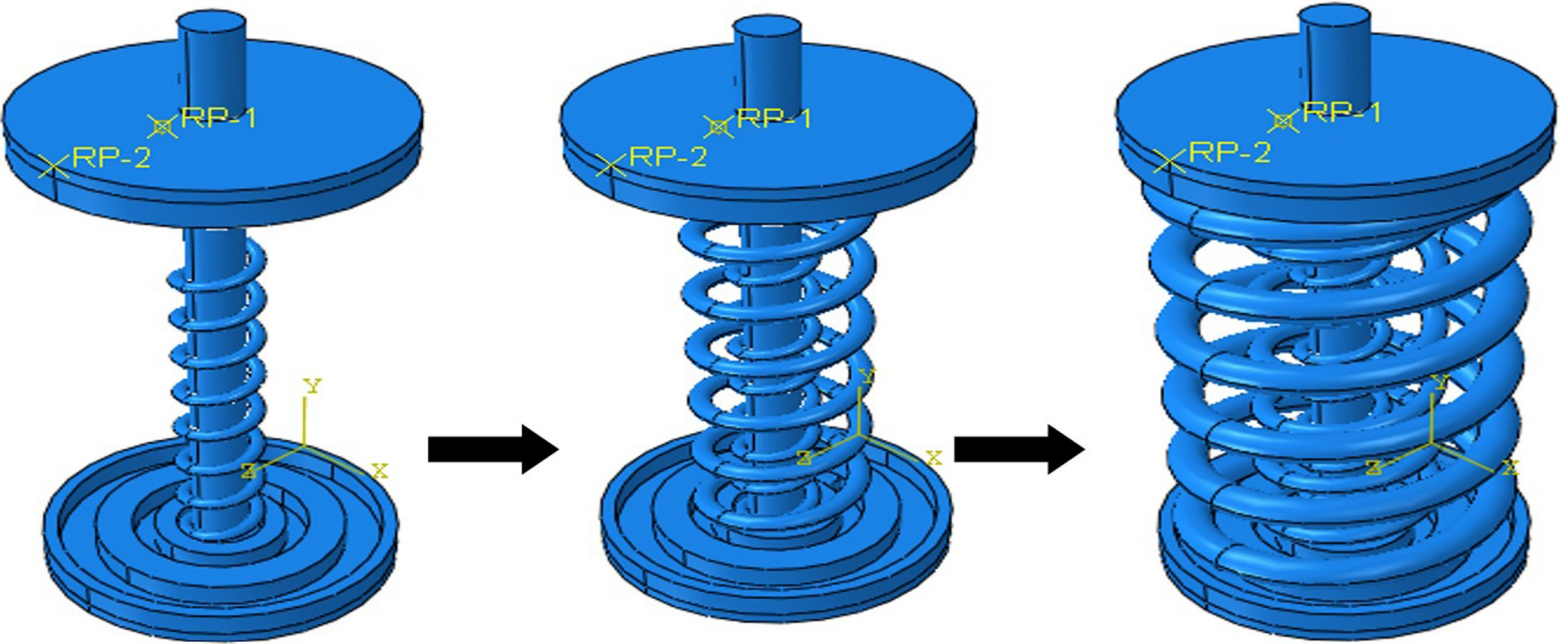
Assembly of AVSR.

A numerical simulation was carried out for the AVSR prototype with the properties mentioned in Table 1. The Variable Stiffness Restrainer has been drawn in SOLIDWORKS software [27] and imported to ABAQUS CAE [28] as illustrated in Fig 3. The elastic steel properties are shown in Table 2.

**Table 1:** Configuration details of AVSR prototype.

Model	d_1_	d_2_	d_3_	D_1_	D_2_	D_3_	L_1_	L_2_	L_3_	n_1_	n_2_	n_3_
**1**	12.5	7.5	5	110	60	35	200	170	150	5	5	7

*Units in millimeter except n is unitless

**Table 2:** Steel elastic properties [29].

Prototype	Density kg/m^3^	Young modulus (GPa)	Poisson’s ratio
AVSR	7800	193	0.3

**Fig 4:**
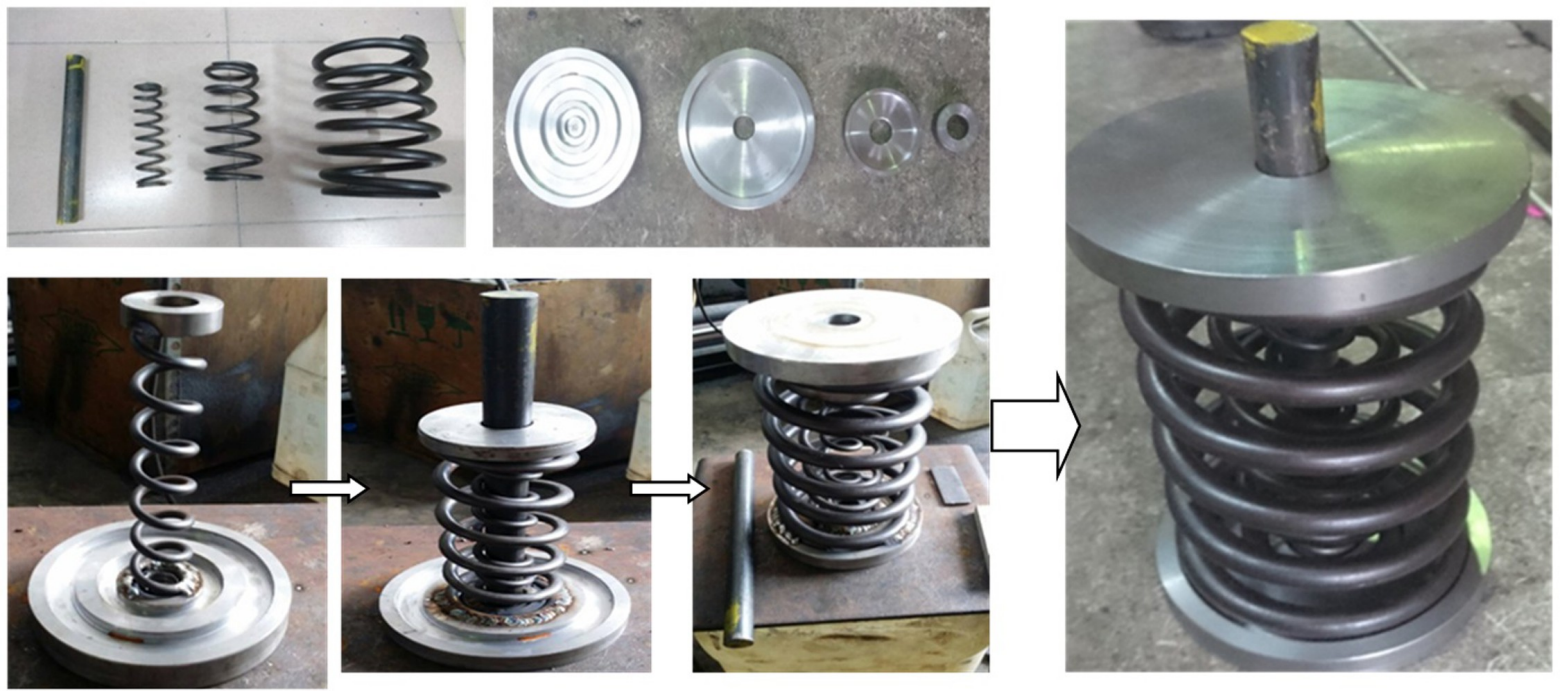
The fabrication of AVSR prototype.

The purpose of an experimental study is to verify the structural characteristics and efficiency of proposed restrainer. One small prototype was fabricated then; incremental and cyclic loading have been performed on the AVSR using dynamic actuator with 300kN load capacity to evaluate the cyclic behavior of AVSR, the ultimate strength and displacement of the restrainer. The fabrication of AVSR prototype was conducted in four steps as shown in Fig 4. In the first step, the shaft and springs have been fabricated. Second, the plates were provided and turning process has been implemented on each plate to form the covers needed to be fitted in each spring. The four different widths of support positions in the lower cover in order to put the springs and the shaft. However, equal size holes (20mm) have been drilled in upper covers to enable the shaft pass through them. Next, the inner spring has been welded to the upper and lower cover. After that, the middle spring was welded to both upper and lower cover. By repeating the same process with the outer spring, the restrainer shape will be formed. In the last step, the shaft has been added to the AVSR for springs movement control. The shaft welded only to the lower cover while upper covers are freely attached to the shaft forming the prototype of the device.

### Setup and testing of AVSR

**Fig 5:**
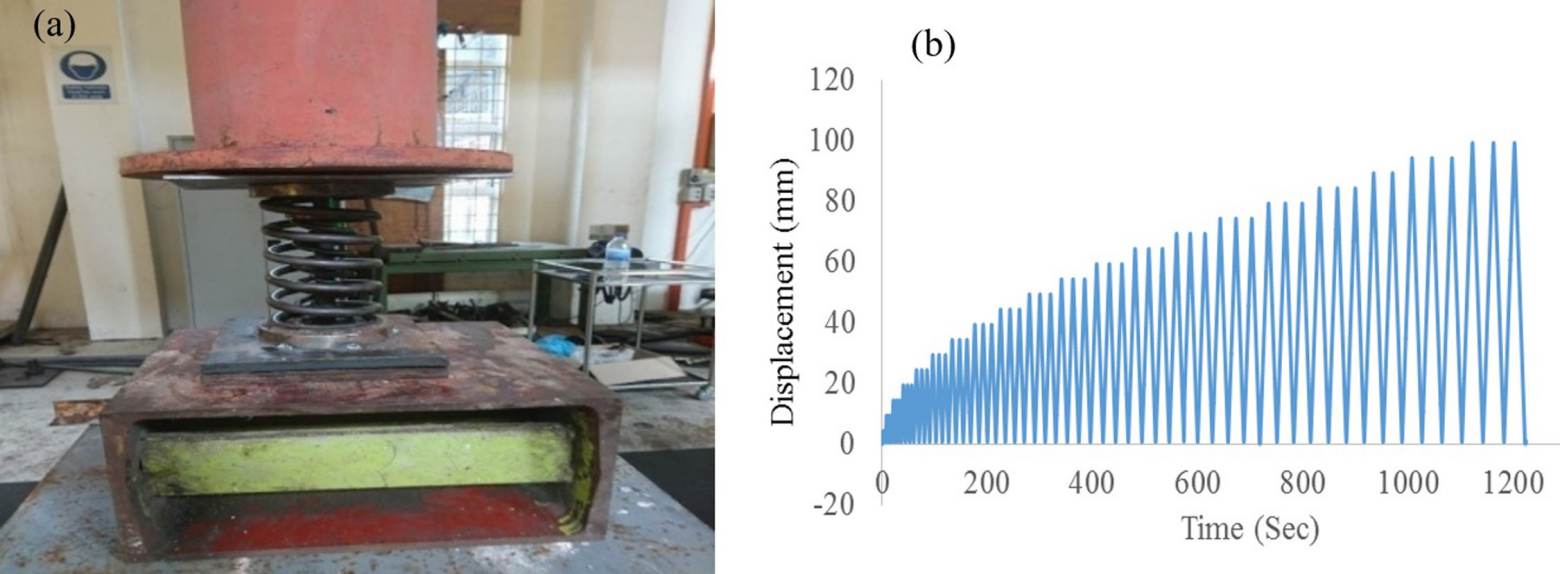
(a) Experimental setup for AVSR prototype (b) Applied time history displacement [30].

The prototype has been tested in UPM structural laboratory and dynamic actuator machine with 300kN testing capacity was used for prototype testing in order to examine the performance of AVSR as the test setup in Fig 5A. Two steel plates were added at the top and bottom of the device to provide rigidity and sliding prevention of prototype as well as transfer the load uniformly from the actuator to the device. The load displacement results have been reported directly to data logger through signal sent from LVDT and load cell located on the actuator. The required maximum displacement for calculating the displacement amplitude of loading histories was considered as 100mm. The pushing test has been conducted to demonstrate the three levels of restrainer functionality. The applied displacement was on top part of the AVSR in a downward direction at a constant rate until getting maximum displacement of 100mm. The cyclic loading test was carried out for the restrainer according to Applied Technology Council [30]. Similar to pushing test, the cyclic displacement applied in the downward direction at the top of the device with 5mm incremental, then upward to show unloading condition. The steps have been repeated until getting 100mm displacement which is the target maximum movement. The considered time rate for cyclic load is 2mm/sec for the first cycle and increased up to 100mm/sec for the last cycle. Fig 5B shows time history cyclic displacement used for test as described.

### Numerical simulation and experimental testing results

**Fig 6:**
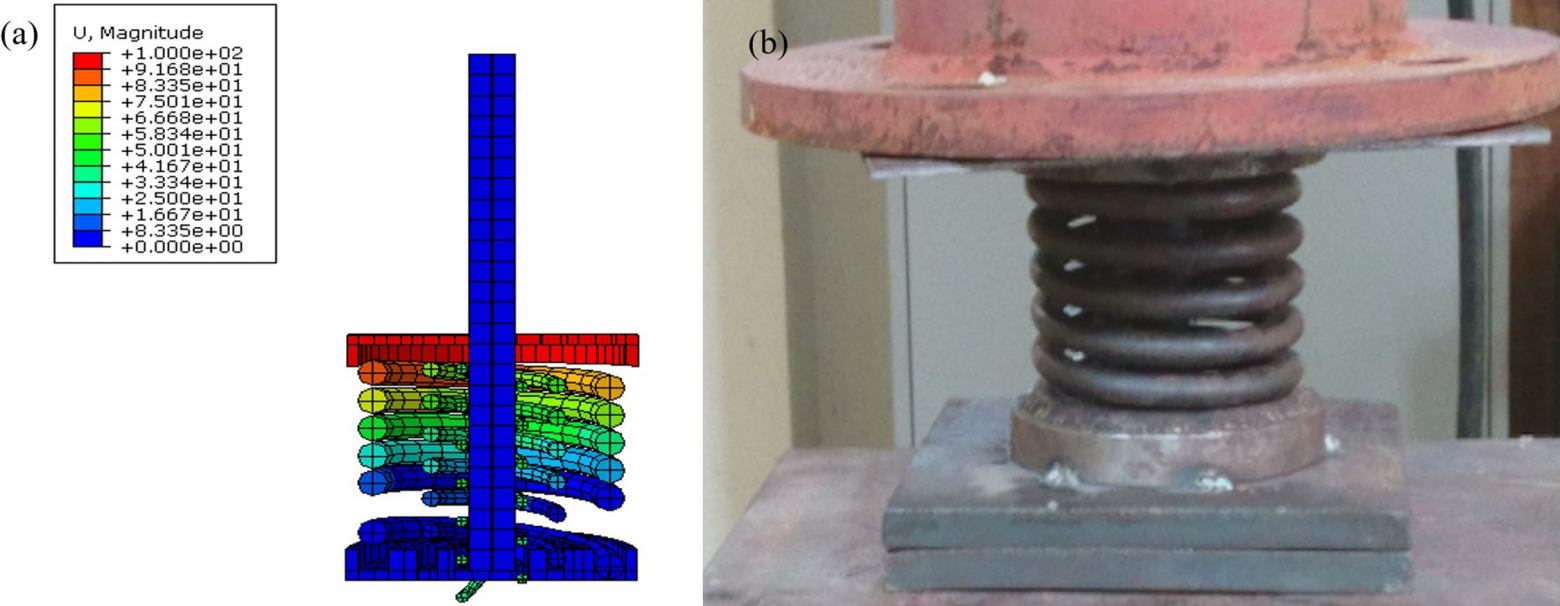
Elastic deformation of AVSR: **(a)** FEM, **(b)** Experimental.

The incremental pushing and cyclic tests have been conducted on AVSR in order to assess function of device in three levels and validate the developed constitutive model for this device. Fig 6, illustrates the device deformation after all the springs have been compressed and the displacement has reached to 100mm which is the maximum allowable movement of the device. Furthermore, the finite element results showed a significant match with the experimental tests for displacement and force resistance for both incremental and cyclic analysis. The results indicated that the device capacity reached to 5.6 kN which is slightly higher than experimental test output. Furthermore, elastic deformation was seen for each spring and the device returned to its original shape after releasing forces.

**Fig 7:**
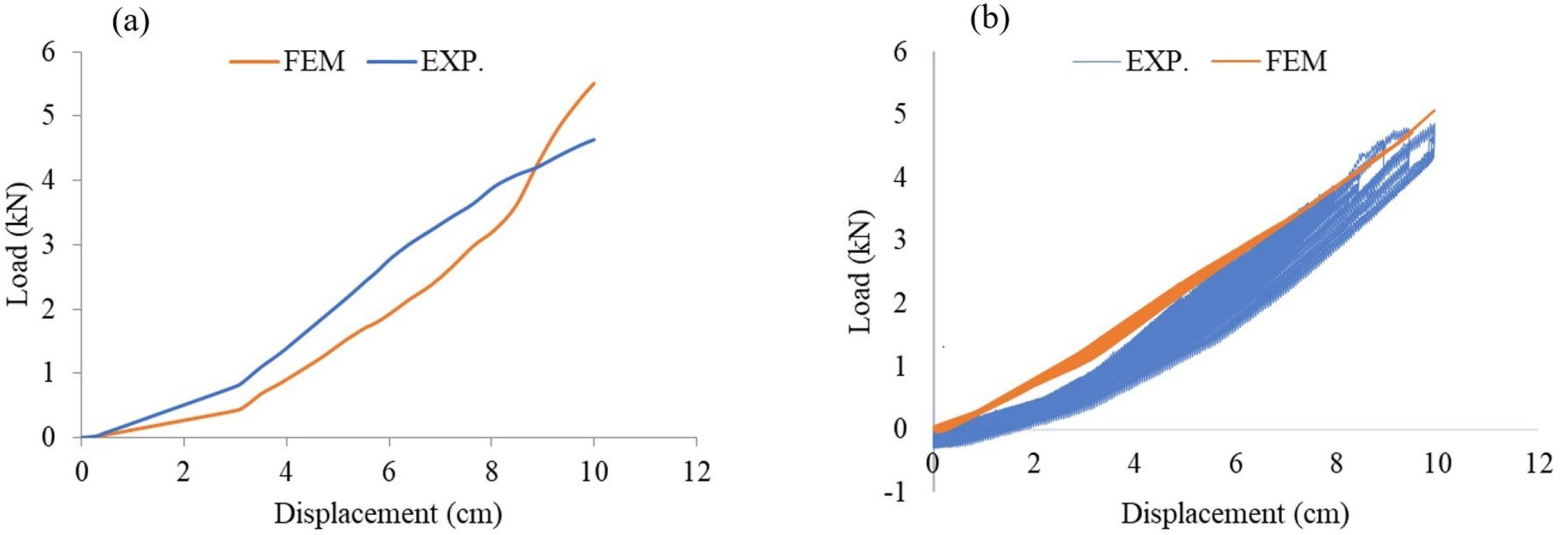
Load displacement curve for AVSR from experimental and finite element model: **(a)** Incremental load, **(b)** Cyclic.

Fig 7 shows the load displacement curve of AVSR for both experimental and FEM incremental load tests. The load displacement behavior at the first level was a linear relationship and the displacement was controlled at 3 cm. After that, the middle spring started working along with the outer spring to show the second level of bilinear elastic behavior and the load rate had increased compared with level one. Thus, the stiffness of the device recorded a suitable incremental when the displacement increased. The third spring began to function with the other springs when the displacement reached 6cm and the device’s workability has improved showing trilinear elastic behavior until achieving 10 cm displacement. Subsequently, all springs reach their solid length and the device functions as a rigid stopper with large stiffness. Fig 7 also shows the comparison between experimental and FEM incremental load analysis results in order to assess the validity of the experimental results. The achieved numerical result demonstrated a good agreement with experimental test and constitutive model. Since, the constitutive model’s maximum force was 5.94 kN. The output of the cyclic testing is the hysteresis forced displacement curve that was experimentally and numerically achieved under half cyclic loading. Since AVSR functions in one-sided movement, only a positive sign is demonstrated in the hysteresis curve. From the curve mentioned, the load and displacement values are the same as in the incremental load test, and the three levels of movement are shown at 3cm, 6cm and 10cm for low, moderate and high stiffness respectively. At the first movement level, the load reached 1.5 kN until the second spring function. Then, the load increased two times to show improvement in the device stiffness behavior. When the combinations of springs work together, device’s structural behavior and stiffness are improved and the maximum load is reached at 5kN. The experimental results were validated with FEM and a constitutive model and the outputs were in line with experimental results. The peak loading for the specimen was about 4.7 kN from the experimental testing. However, 5.6 kN and 5.94 kN were the peak loadings for the specimen from FE and the constitutive model respectively. Therefore, the divergence between experimental, FEM and constitutive model for incremental load and cyclic testing was less than 15% due to the fact that the exact elastic behavior of a steel spring is difficult to achieve during experimental since it may be affected by rust which reduces the spring capacity.

## Parametric study on AVSR specifications

The main purpose of analyzing different configurations of AVSR models is to find the best configuration of AVSR to have the desired performance and validate the derived constitutive model. Also, evaluate the effect of different parameters on the performance of proposed AVSR. To evaluate the effect of spring’s configurations of AVSR, twelve models including different types of devices were modeled and analyzed. The steel material had a shear modulus about 75 GPa in all models, which represents cold-rolled steel that has been used in experimental work as well.

The circular coiled spring is the most common spring type and is widely utilized in different applications. To perform a parametric study for a device with circular wire cross section springs, there are four effective parameters which are wire diameter, spring mean diameter, number of coils and spring length. Therefore, six models with rounded shapes have been chosen with different configurations as illustrated in Table 3. The wire diameter of outer spring (d1) varies from 17 to 30mm to evaluate the effect of this parameter. Similarly, middle spring wire diameter (d2) has been chosen. However, the inner spring wire diameter (d3) has been selected with a smaller diameter due to the difficulty of manufacturing such types of springs.

**Table 3:** Configuration of circular wire coiled type AVSR models.

Model	d_1_	d_2_	d_3_	D_1_	D_2_	D_3_	L_1_	L_2_	L_3_	n_1_	n_2_	n_3_
C1	25	22	20	230	150	80	1000	850	700	6	7	9
C2	30	30	25	240	180	120	900	750	600	10	9	8
C3	20	22	25	260	220	176	600	450	300	6	7	7
C4	22	25	25	180	136	86	800	650	500	8	7	8
C5	20	20	25	210	170	130	1100	950	800	8	9	7
C6	17	15	22	175	141	111	850	700	550	8	8	8

*Note: Dimensions in mm except n is unitless

The mean spring diameter of outer spring (D1) has been selected in range of 175mm to 260mm to enable the other two springs to be located inside the outer spring while D2 and D3 should be calculated. The diameter of middle spring (D2) is equal to D1-2*d1, while the inner spring diameter (D3) is equal to D2-2*d2. The device length depends on the outer spring free length (L1). However, the other two spring free lengths should be decided based on the displacement needed for each level of movement. In other words, free length of middle spring (L2) is equal to L1-δ1 and δ1 is the working range of the first stage of AVSR to allow outer spring to operate alone. Nevertheless, free length of inner spring (L3) can be calculated by L1-δ2 where δ2 represents the device’s second operation displacement. The working operation range has been decided to be 150mm for each stage and the maximum displacement assigned to be 450mm. Therefore, device length varies from 0.6 to 1.1m. The number of coils is decided according to free length and solid length needed for each spring. The configurations of device with rectangular coiled springs have some similarities with the specifications of circular coiled spring such as free length selection or calculation and number of coils. However, the main difference between them is wire cross section. Die spring with a rectangular cross section have the advantage of demonstrating higher force than circularly coiled spring with same working operation range. Table 4 illustrates six model configurations to be analyzed and evaluated.

**Table 4:** Configuration of rectangular wire coiled type AVSR models.

Model	a_1_	a_2_	a_3_	b_1_	b_2_	b_3_	R_1_	R_2_	R_3_	L_1_	L_2_	L_3_	n_1_	n_2_	n_3_
D1	25	22	25	30	25	30	110	85	63	650	550	450	7	8	8
D2	25	20	20	30	25	25	130	105	85	750	650	550	6	7	7
D3	25	25	25	40	40	40	90	65	40	850	750	650	9	9	10
D4	20	20	20	40	40	40	80	60	40	900	800	700	10	11	11
D5	25	25	20	25	30	30	125	100	75	700	600	500	6	7	7
D6	20	20	10	30	30	25	110	90	70	700	600	500	8	8	8

*Note: Dimensions in mm except n is unitless

The wire widths of outer (a1), middle (a2) and inner (a3) spring were selected between 10 to 25mm.While, the selected range for wire height for the three springs mentioned b1, b2 and b3 was between 25 to 40mm. The mean radius of outer spring (R1) selected to vary from 80 to 130mm to ensure that the other two springs are easily placed. However, R2 should be calculated to be R1-a1 and R3 equal to R2-a2. The device length depends upon outer spring free length (L1) and the other two spring free lengths should be decided according to the displacement required for each level of movement and device length vary from 0.65 to 0.9m. The working operation range for this type of device was 100mm for each phase and the maximum displacement was 300mm since the die spring working range is less than that of a circularly coiled spring. The coefficients ¥1, ¥2 and ¥3 were calculated using Eq 15 which depends on the wire section dimension. The number of coils is decided according to free length and solid length needed for each spring.

**Fig 8:**
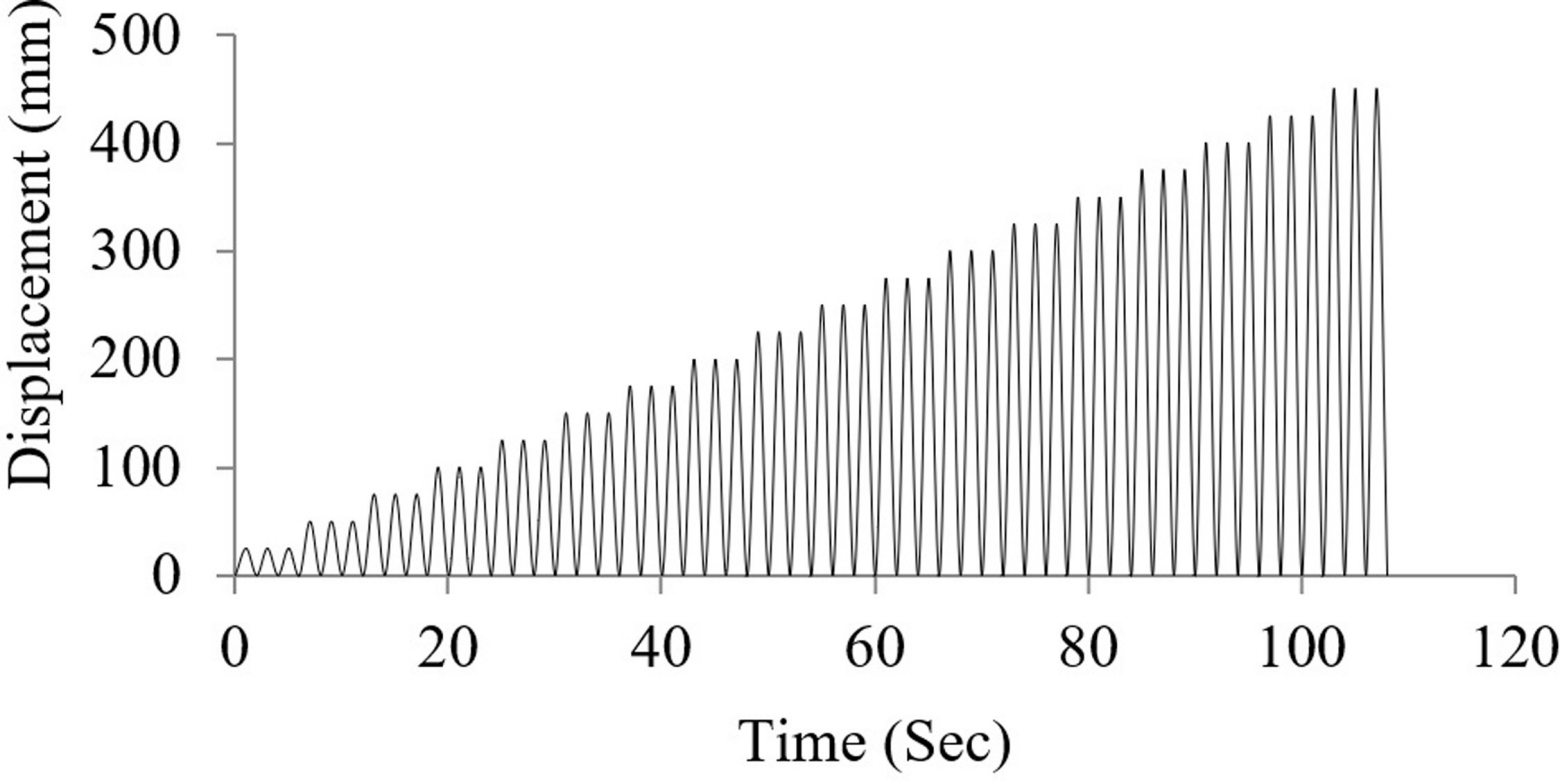
Cyclic load applied on AVSR.

For FE incremental loading analysis, the displacement was applied as a push until getting maximum displacement. For cyclic loading, the displacement was applied as push and release protocol by applying it on the upper cover in the downward direction demonstrated in Fig 8. The considered maximum displacement of circular and rectangular wire type AVSR is 450mm and 300mm respectively since die spring is stiffer and demonstrates higher resistance than a normal circular wire spring with same displacement range. In addition, the rectangular shape will restrict the spring’s movement in axial direction and less operation range will be allowed. For boundary conditions, rigidly fixed geometry was used at the bottom of the device.

### Results and discussions of parametric study on AVSR specifications

**Fig 9:**
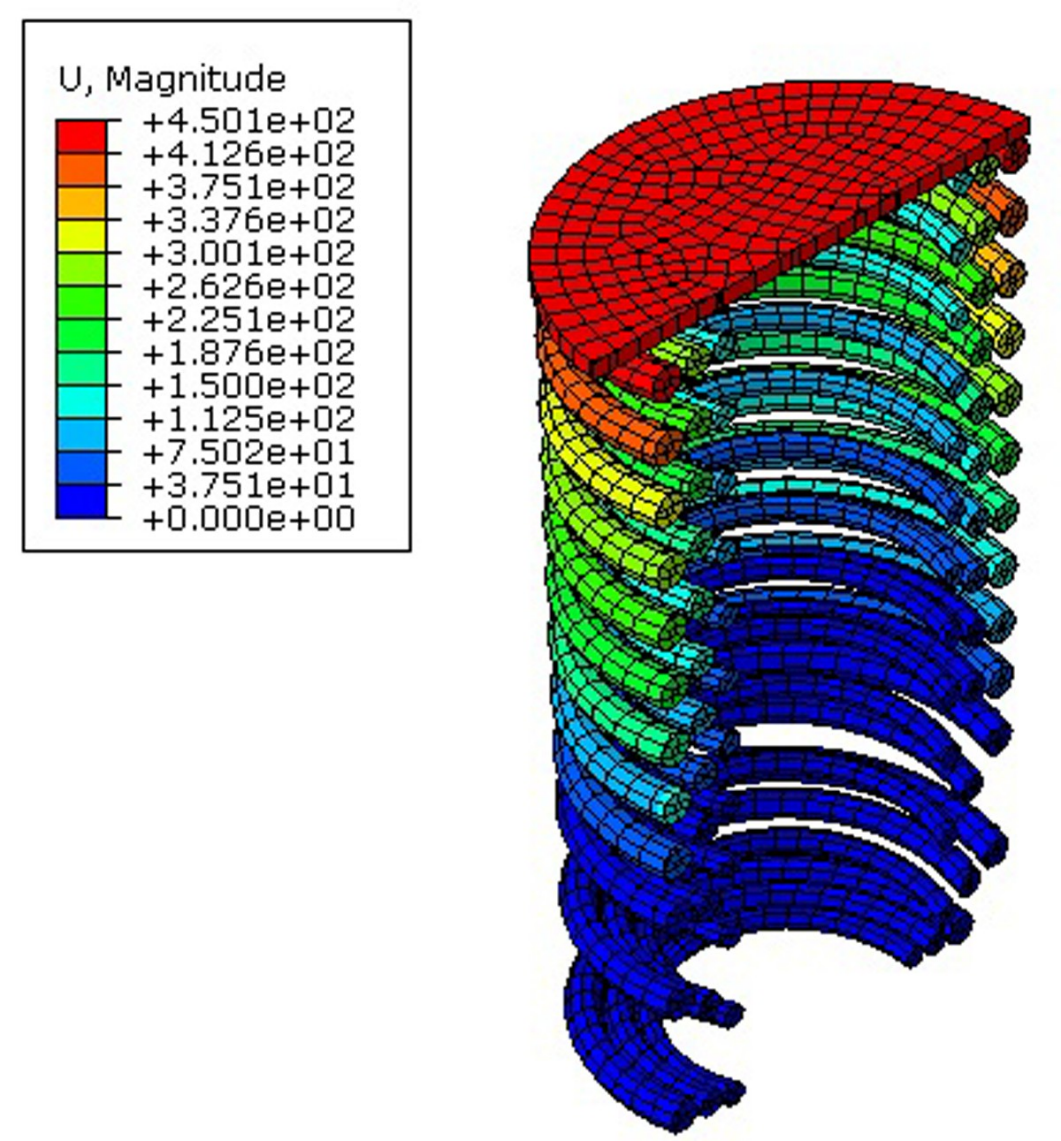
Numerical displacement results for model C1.

AVSR performance is affected by spring properties, wire section diameter, mean radius or diameter, number of coils and maximum deformation. Therefore, a parametric study was performed to assess the impact of each parameter on the workability of the device. For the six circular wire AVSRs, the displacement limit was chosen to be equal for every stage of movement. The results revealed that the load capacity of devices with rectangular wire was higher than AVSR with circular wire and there was less displacement, indicating higher stiffness and better performance. The trilinear behavior of AVSR was seen in all 12 models during incremental load and cyclic tests. In the first stage, linear elastic behavior was exhibited by the devices. Then, bilinear elastic behavior was shown in the second step. Lastly, the trilinear behavior was achieved by the end of stage three. These results demonstrated that the device’s multilinear elastic behavior is not affected by varying the AVSR parameters. However, these characteristics will affect device stiffness and capacity in each stage. Fig 9 illustrates the finite element results for first model of the circular wire type AVSR (C1).

**Fig 10:**
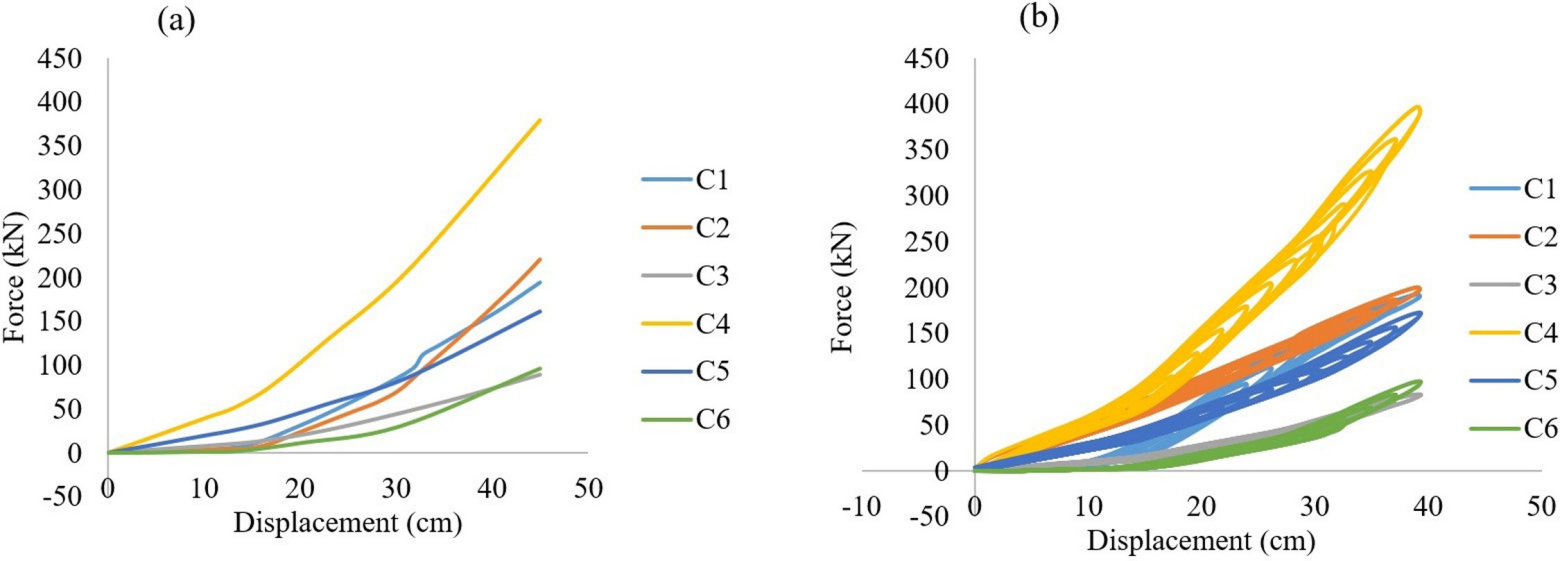
**(a)** Force displacement incremental curves **(b)** Hysteresis behavior of different circular wire type AVSR.

The output of incremental load and cyclic analysis showed the three-level behavior of proposed device. Fig 10 illustrates the load displacement curves for the six circular wire AVSR. Model C4 showed the best performance among all the models in all stages, the capacity was around 70 kN in the first step. Moreover, model C5 demonstrated about 30kN while the remaining models capacity during first stage was less than 13kN. The resistance of the device has improved in the second step and device model C4 still demonstrating highest performance with a capacity of about 200kN. However, the model C6 force was about 30kN which is the lowest device resistance due to low wire diameter of the second spring in model C6.

Furthermore, model C1 and C5 loads had almost the same resistance in the second stage with around 75kN which indicates model C1 performance has considerable improvement while transferring to the second step due to low mean diameter and number of coils of second spring compared with first spring in model C1. In the third level, model C2 recorded the high load incremental and reached 220 kN since the largest wire diameter for all springs was in model C2 compared with other models. In contrast, model C3 demonstrated the lowest incremental resistance of about 89 kN due to large mean diameter of the model C3 springs. In addition, model C6 performance was slightly better than that of model C3 with 96 kN resistance because of low wire diameter of each spring in model C6. Model C1 load incremental performance was better than that of model C5 and the peak capacity was 194 kN while 161 kN was the peak resistance of model C5 by the end of stage three due to the characteristics variation between them in terms of number of coils and mean diameter for each spring in both models. As mentioned above, device model C4 showed highest device resistance by the end of the last stage and it was about 380kN. As same incremental load analysis, cyclic tests were performed on the six circular wire type AVSR models. All the mentioned models demonstrated stiffness incremental while transferring between levels since the first spring started to function at the beginning of the first stage and then the middle spring was involved to improve the stiffness of the device. By the displacement incremental, the device stiffness has increased since the third spring has also been involved to AVSR performance. However, smooth stiffness degradation has been shown while unloading each model which makes the hysteresis behavior nearly linear, demonstrating elastic behavior of AVSR numerically. The results of dynamic tests were almost same static tests in terms of load resistance with a slight difference between them. However, the displacement limit was slightly less than static tests since dynamic test is faster and the transferring between levels required less displacement. Furthermore, the three level behaviors has also seen in cyclic test and model C4 showed the best capacity with around 397 kN which is less than 5% difference from static force result. While model C3 demonstrated 83kN force resistance which is the lowest cyclic resistance among all and around 6% less than static resistance for model C3. Moreover, model C2 showed around 9% variation between static and dynamic tests while the remaining models demonstrated negligible difference between them.

**Fig 11:**
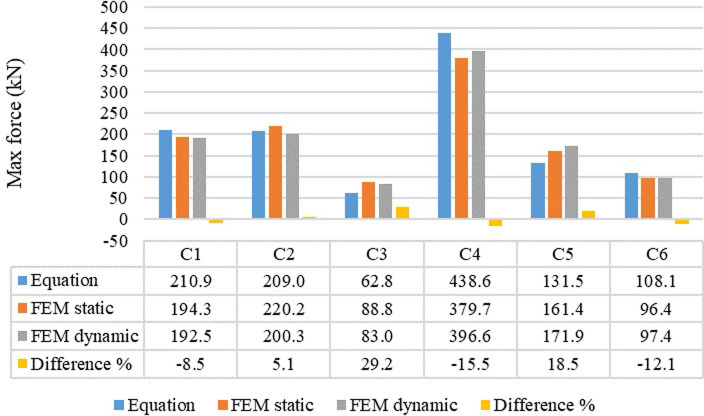
Comparison between constitutive model and FE results for different circular wire type AVSR.

The circular wire type AVSR finite element results have been compared with the derived constitutive model to validate the FE outputs as illustrated in Fig 11. The variation was less than 20% for five models between FE and equation. However, only model C3 resulted in around 29% difference. The reason behind these variations was due to the fact that in finite element analysis the device is modeled as full geometry and the performance is affected by different test parameters such as material properties, mesh, and boundary conditions. Though the device performance in the constitutive model depends on direct input effective parameters that will not be affected by abovementioned test parameters.

**Fig 12:**
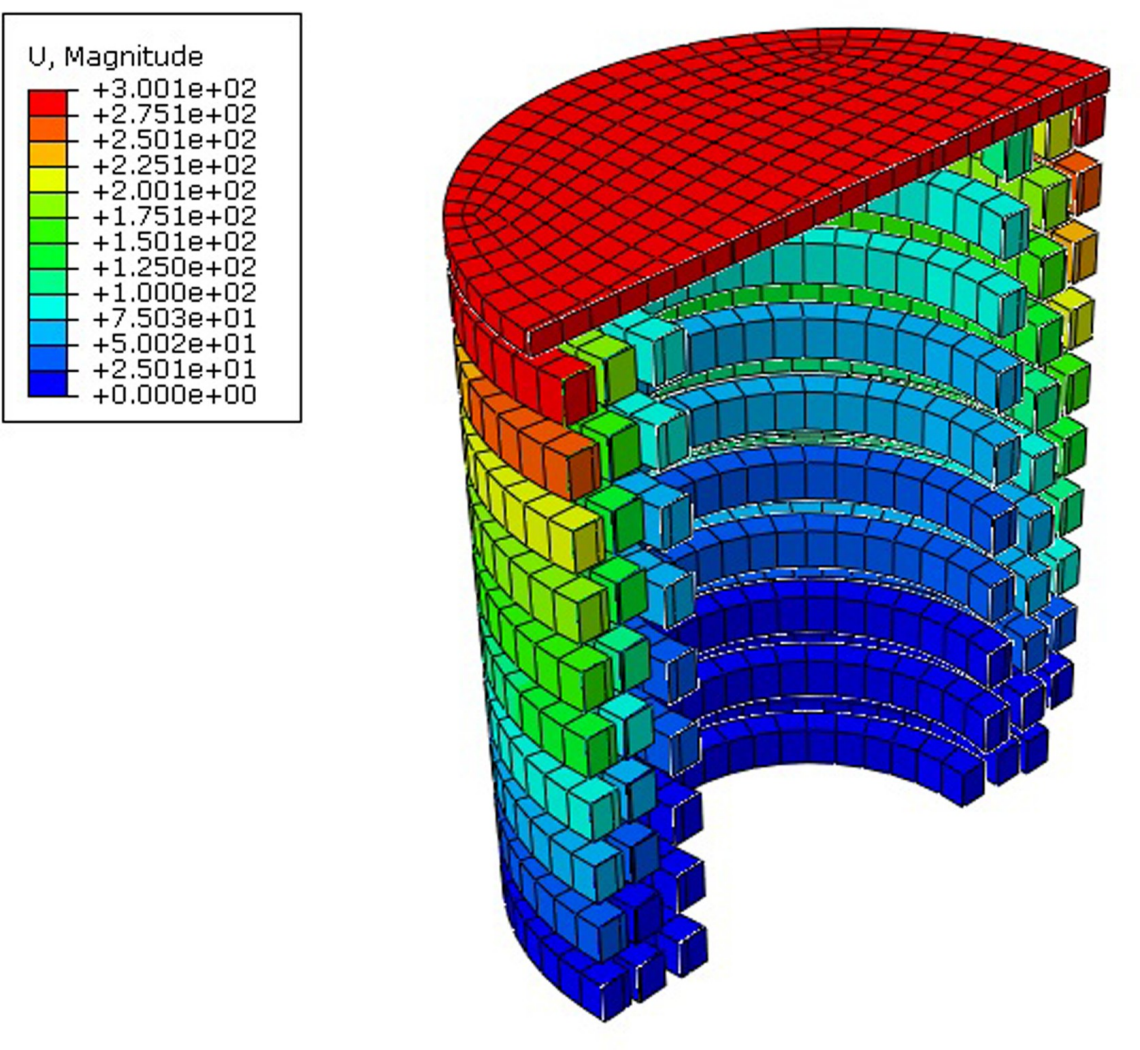
Numerical displacement results for model D1.

The outputs of rectangular wire type AVSR finite element models showed better performance, load resistance and stiffness compared with circular type AVSR. Fig 12 illustrates the deformation for first rectangular wire type AVSR (D1). Model D3 has the best device performance and the maximum force was about 1069 kN which is significantly higher than other devices capacities because of largest wire section area, highest wire height/width ratio and smallest mean radius of each spring compared with other models. However, models D2 and D6 showed the lowest resistances with about 63kN and 59kN correspondingly due to their low wire section area, large mean radius, and low wire height/width ratio for every spring in mentioned models. In addition, Model D4 exhibited high capacity with around 605 kN since the mean radius of each spring was considerably low with large number of coils.

**Fig 13:**
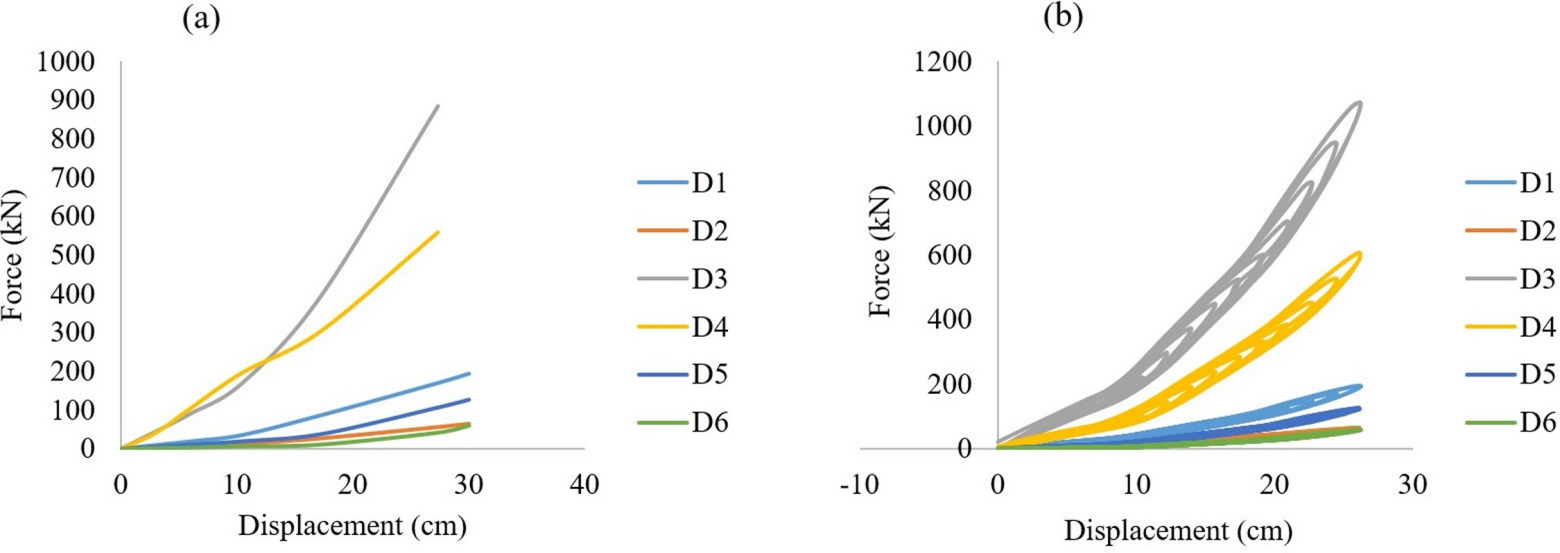
**(a)** Force displacement curves **(b)** Hysteresis behavior of different rectangular wire type AVSR.

Models D1 and D5 have almost the same characteristics. They showed maximum forces of about 194kN and 125kN respectively, due to the difference in mean radius of each spring. As a consequence, the spring mean radius essentially represents the main parameter that has an impact on the rectangular wire type AVSR. Furthermore, the spring wire height, width and wire height/width ratio have a significant effect on AVSR. Additionally, by reducing number of coils the device’s resistance will be improved. Spring length, on the other hand, has not had the same effect on device performance as a circular wire type AVSR. Fig 13 shows the load displacement curves for models D1 to D6 acquired from static analysis. Same as C1 to C6, the multi-level behavior has been clearly seen in models D1-D6 and stiffness has significantly improved during transfer between stages. In the first level, all AVSR models have low resistance except models D3 and D4 have about 170kN and 190kN forces respectively due to significant properties in outer spring in both models. Moreover, model D3 and D4 still have considerable resistance compared with other models during second stage and model D3 reached around 500kN while model D4’s force improved to be about 350kN.The remaining models capacities were less than 100kN. By the end of third level, all models had reached their maximum forces with the same variation between them. In other words, models D3 and D4 still show the best performance while model D6 demonstrates the lowest resistance. As determined by the same incremental load analysis, the trilinear behavior of the proposed restrainer was shown in cyclic test results with almost the same capacities. All the above-mentioned simulations showed improvement in stiffness during shifting between stages while the stiffness regularly decreased during unloading.

**Fig 14:**
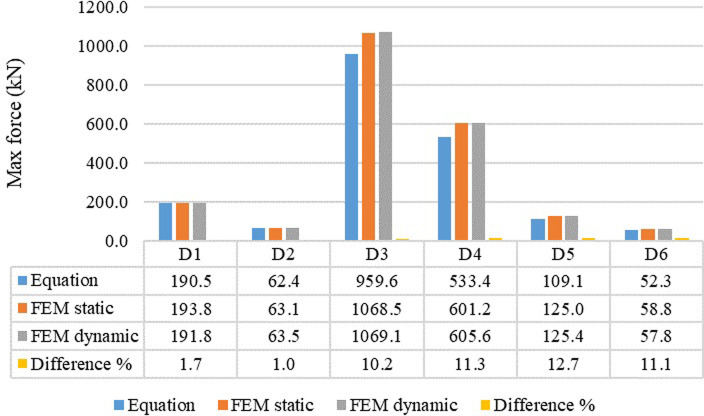
Comparison between constitutive model and FE results for different rectangular wire type AVSR.

The rectangular wire type AVSR finite element analysis outputs were compared with the derived constitutive model to validate the FE results, as shown in Fig 14. The difference was not more than 13% for all models between the FE and mathematical models. The models D5, D4 and D6 resulted in variations between FE and constitutive model of about 12.7%, 11.3% and 11.1% respectively, while the remaining models showed less difference. These differences were similar to the circular wire type AVSR, because the AVSR simulated complete geometry and the performance is affected by different test parameters such as material properties, mesh, and boundary conditions in FE analysis. However, the device capacity and stiffness in mathematical model depend on direct input effective factors that will not be affected by the abovementioned test parameters.

To sum up, the outputs revealed that by increasing the spring section area, the device capacity will be improved. In other words, the resistance will be enhanced when the wire diameter increases for a circular wire spring. However, for springs with rectangular wire, incremental wire height and width increased capacity. Additionally, increasing the percentage between width and height will enhance the AVSR stiffness. On the other hand, increasing the spring’s outer diameter and number of coils will result in a reduction in AVSR force resistance. Moreover, each spring length has no effect on the device’s stiffness or capacity. However, the length has a considerable impact on number of coils selected for every spring. At the same time, device total length depends on outer spring free length, which should be selected carefully in order to attach the AVSR to the bridge correctly. The results also showed that device maximum displacement essentially depends upon each spring operation range. Hence, the AVSR will stop movement and convert to a rigid stopper when any spring reaches its solid length. Therefore, the displacement limit for each spring should be assigned with respect to other springs in order to get the desired multi-level behavior for different bridge cases.

## Numerical analysis procedure of frame equipped with AVSR

The numerical procedure for a bridge frame equipped with the proposed restrainer is presented. The numerical analysis is conducted based on single degree of freedom (SDOF) concept. Time history analysis for different frames, including frames without and with AVSR was performed under El-Centro, (KOSK SAGLIK OCAGI) Turkey, Chi-Chi and Kobe excitation’s records. Additionally, the AVSR geometry specifications have been varied to predict the structural response with different parameters that have a highly pronounced effect on the device response. The dynamic performance of a developed AVSR was assessed by means of Newmark’s technique [31] which was codified using a MATLAB program.

### Single degree of freedom system with AVSR

**Fig 15:**
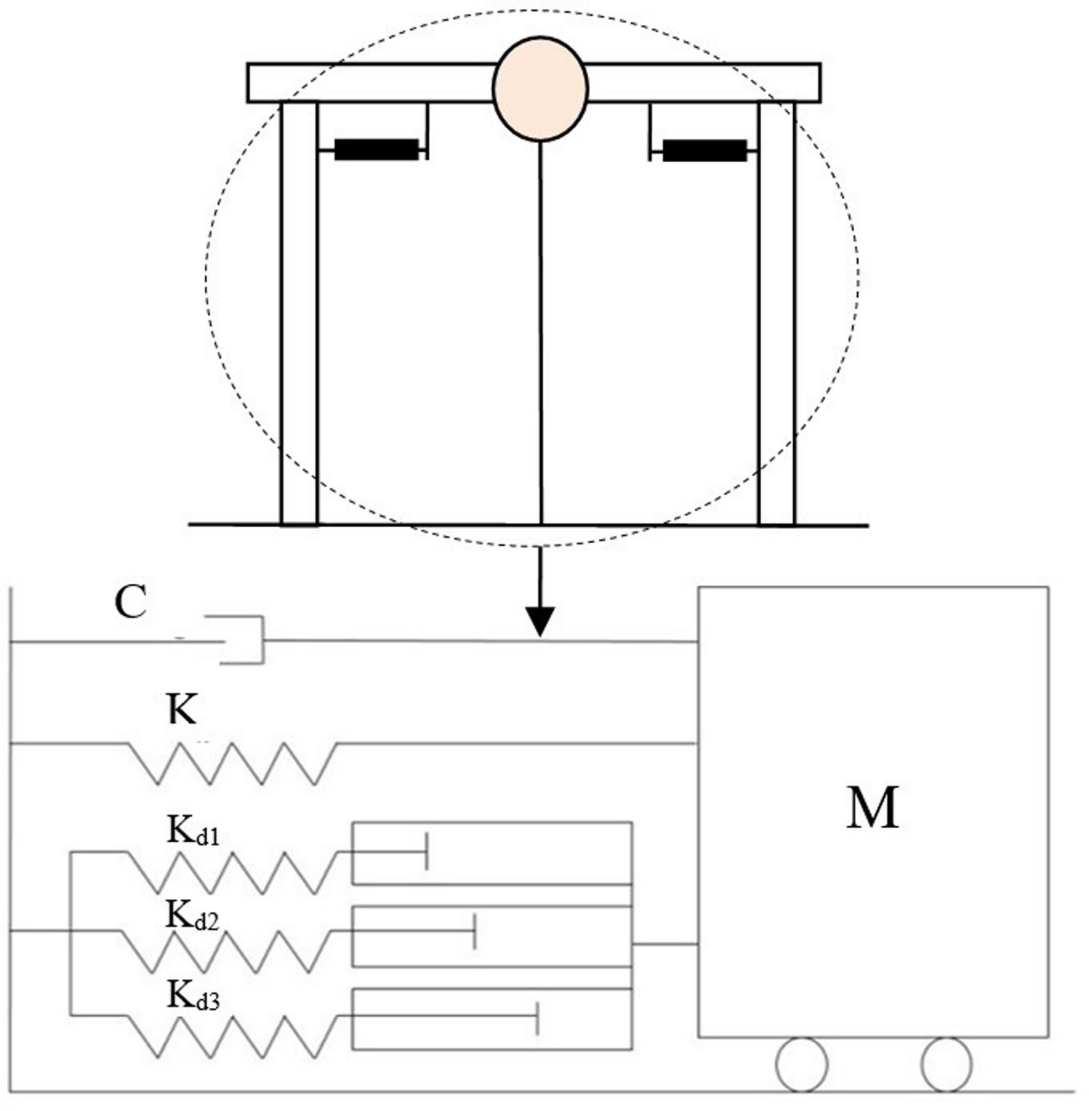
Single degree of freedom system of bridge frame with AVSR.

A showed in Fig 15, the bridge is considered as Single Degree Freedom System (SDOF). For this purpose, the span of bridge is considered as lumped mass (m), the bridge piers are considered as spring of SDOF system, and a pair of AVSR devices are considered as supplementary variable stiffness system (K_AVSR_). Damping of bridge (C) can be determine through conventional method of Rayleigh damping or determine through considering 5% damping ratio for RC bridges and 2% for steel bridges. Then the general equation of motion for single degree of freedom systems is implemented as Eqs (22) and (24).


$$m.\ddot{x}+c.\dot{x}+k.x=f(t)$$ (22)

Where, t refers to the time and the solution will be achieved for a particular input f (t) with given structural characteristics, and f (t) is the applied dynamic load such as a wind or seismic event.


$$f(t)\;=\;-m.{\ddot{x}}_{g}(t)$$ (23)

Once, the Variable Stiffness Restrainer is applied to the SDOF model, the model response will be affected since the AVSR made changes to the equation (effective stiffness). The stiffness of a developed restrainer varies in its nonlinear action. The new displacement value will be gotten and replaced in the next step after the end of each loop. Then, the value of top node displacement (u_i_) should be compared with AVSR displacement in order to find the stiffness at each step.


$$m.\ddot{x}\;+\;c\cdot \dot{x}\;+\;\left(k_{AVSR}+k\right)\cdot x\;=\;f(t)$$ (24)

**Fig 16:**
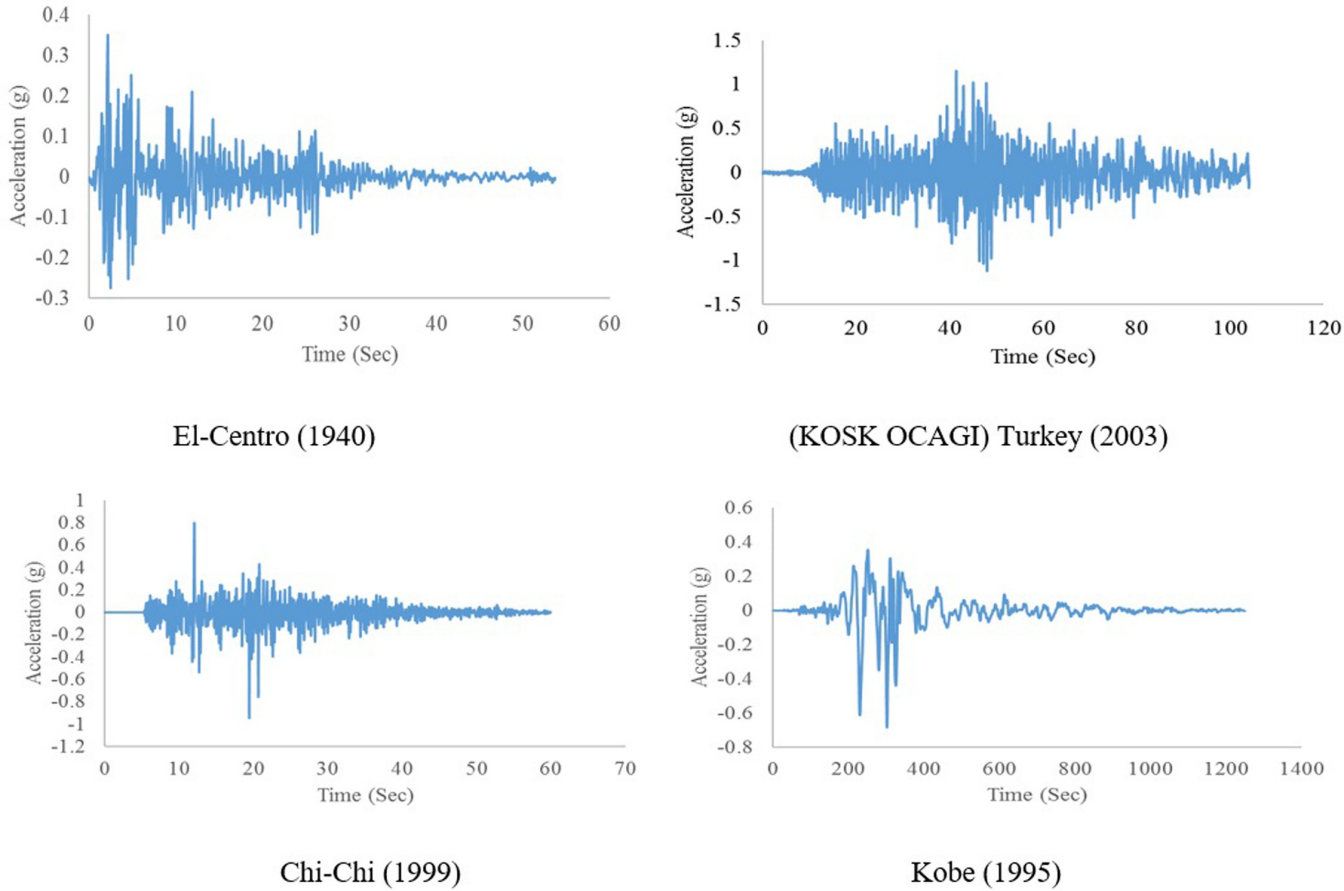
Applied earthquake records.

**Fig 17:**
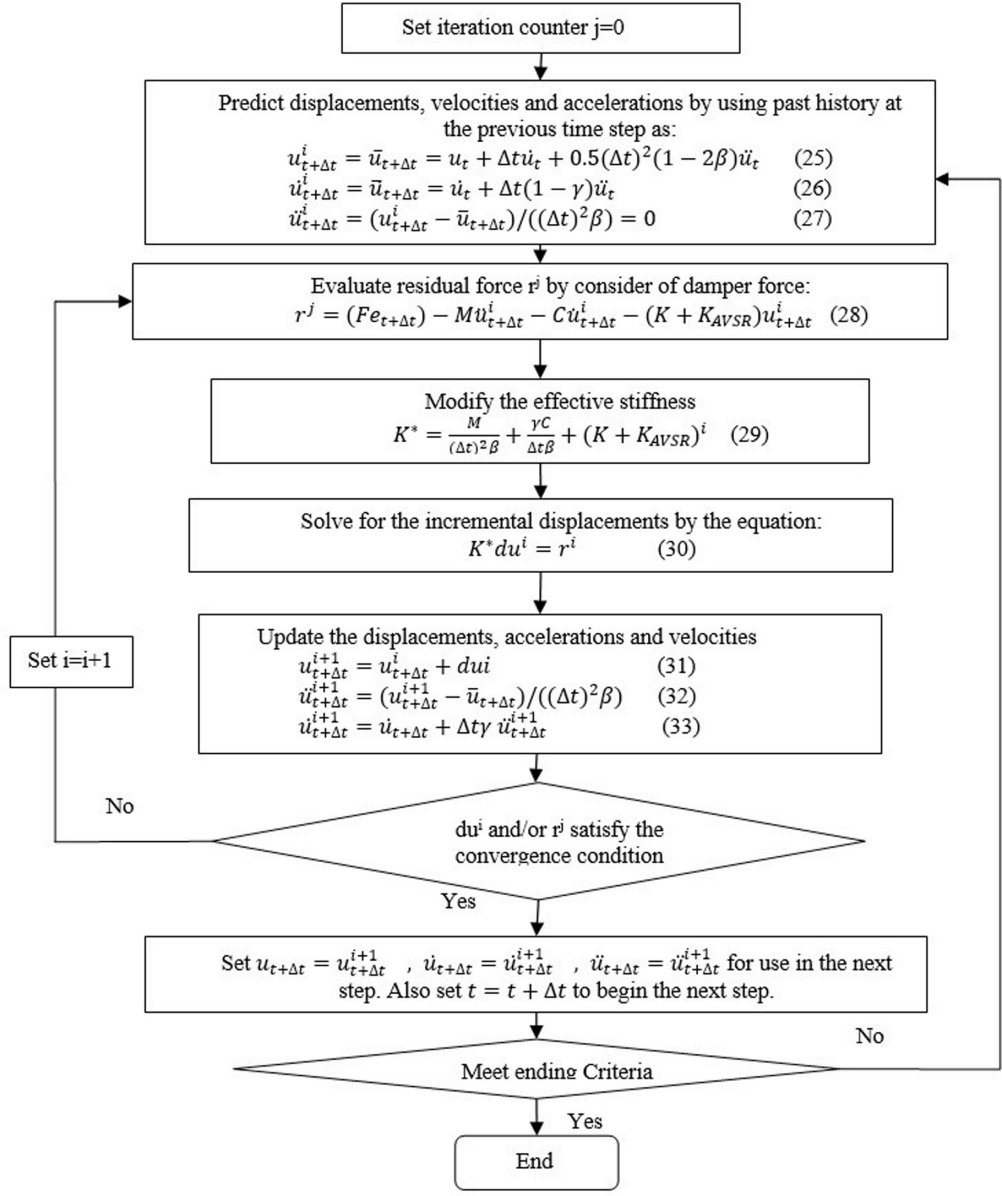
The Newmark’s algorithm for frame equipped with AVSR.

K_AVSR_ represents the stiffness of variable stiffness restrainer. The time history of the El-Centro seismic record, Turkey, Chi-Chi and Kobe considered as input excitations in code as illustrated in Fig 16. The purpose of considering different earthquakes is to demonstrate performance of the bridge frame equipped with developed AVSR system under earthquakes with various PGA. Fig 17 shows Newmark’s algorithm for a bridge frame equipped with a variable stiffness device.

### Results and discussions for time history analysis using Newmak’s method

Assessment of the proposed AVSR efficiency on bridge response subjected to El-Centro, severe Turkey excitation, Chi-Chi and Kobe earthquake time history in terms of displacement, velocity and acceleration was conducted with SDOF system using Newmark’s method in order to assess the effect of the ground motion intensity and duration on the structure’s performance without and with the developed AVSR. The values of mass, damping, and stiffness of the evaluated bridge frame were same in all models. Furthermore, time step was about 0.02 in all selected ground motions.

**Fig 18:**
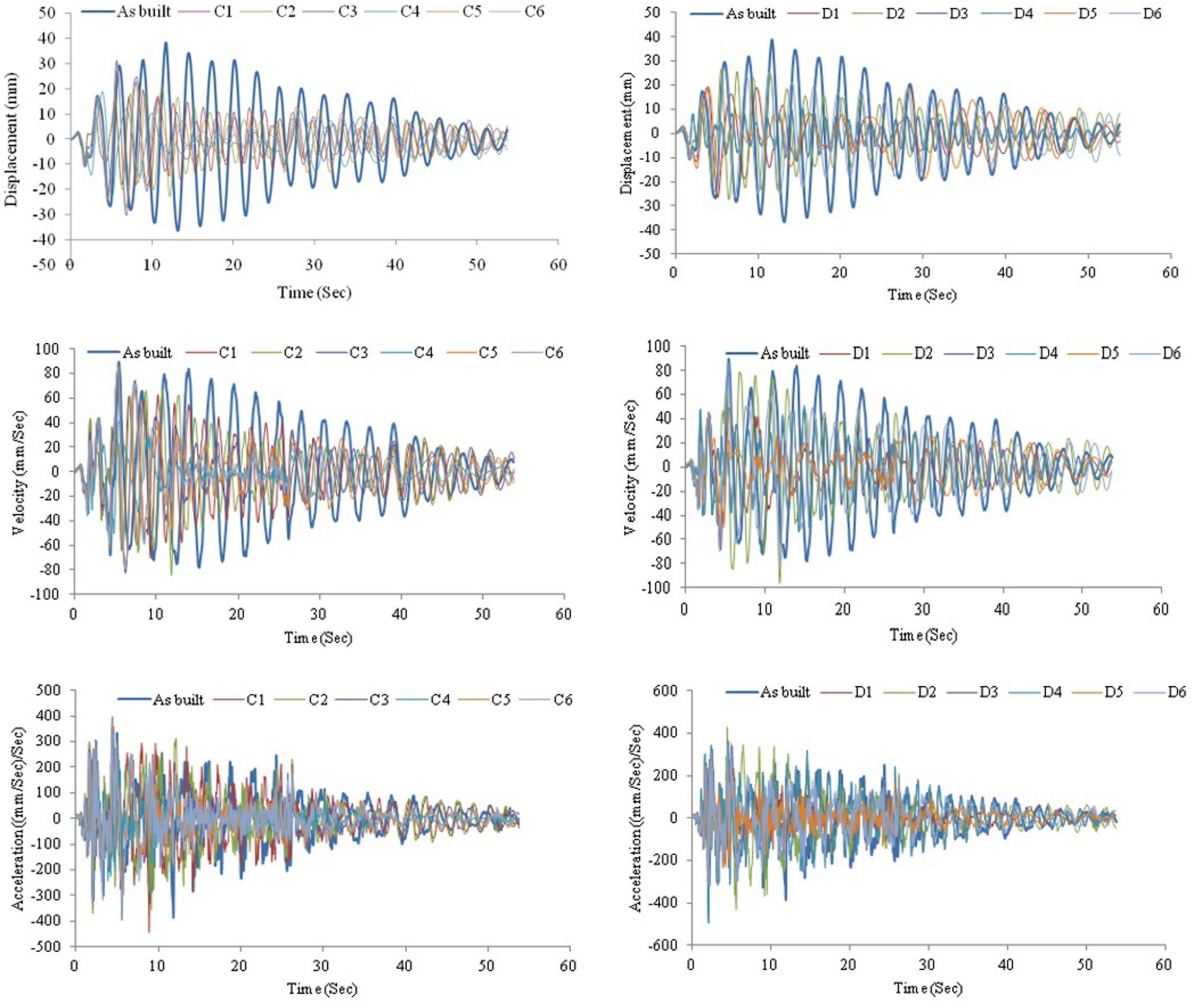
Structural responses for frame without and with C and D device models under El-Centro excitation.

Fig 18 demonstrates the displacement, velocity, and acceleration for a frame without and with AVSR models C and D subjected to the El-Centro earthquake. The circular wire type AVSR is efficient in frame displacement reduction and the declination was varied between circular wire type devices. The peak displacement of bare frame was about 38mm. Equipping the frame with C4 AVSR reduced the displacement by about 50% which is the maximum displacement reduction achieved with circular wire type AVSR models. Furthermore, frame models C1 and C2 reduced the maximum absolute displacement by roughly 48% and 45%, respectively. While, frame displacement has declined by about 18%, 22% and 24% by adding models C3, C6 and C5 of AVSR correspondingly. Frame displacement response has also improved by equipping the frame with a rectangular wire type AVSR. The maximum displacement decrease was around 74% by adding models D3 and D4, while models D2 and D6 demonstrated lower displacement declines of about 31% and 30% respectively. However, the displacement reduction was significantly better than some of circular wire types of AVSR. In other words, die spring type device is slightly more effective in diminishing the vibration effect of El-Centro earthquake. The frame velocity response was also examined by adding AVSR. The maximum absolute velocity reduction was 54% for frames equipped with D5 model compared with a bare frame. In addition, velocity decline varied from 3% to 52% for the other models due to the capacity and stiffness differences between them. In general, frame equipped with D1-D6 models showed more significant reduction in velocity than C1-C6, as a consequence better performance against El-Centro ground motion. The acceleration response for frame without and with different geometry AVSR subjected to El-Centro ground motion was also reported. The absolute maximum acceleration has declined around 17%, 16%, and 15% with models D5, D1 and C4 correspondingly which represents the most significant acceleration reductions among models. Additionally, the acceleration response of frame equipped with C1, C2 and D4 models has reduced by around 8%. Though, frame with model D6 have shown negligible acceleration response improvements of about 1%. In contrast, frame with C3, C5 and D2 demonstrated acceleration incremental by 11%, 2% and 20% respectively.

**Fig 19:**
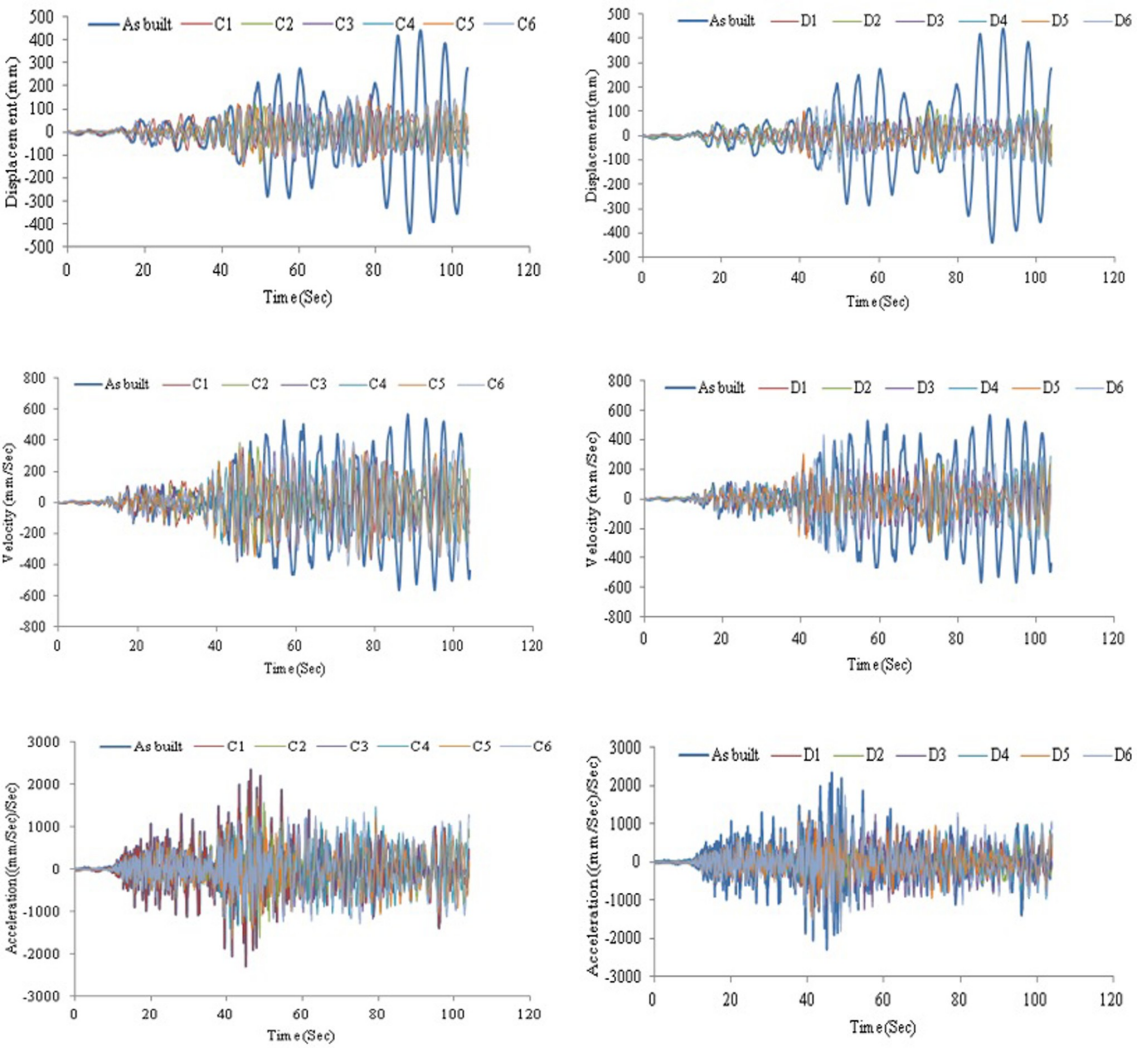
Structural responses for frame without and with C device model under Turkey excitation.

AVSR efficiency has also been evaluated with severe turkey ground motion. The intensity of this earthquake is greater than 1g and its duration is above 100 seconds. The results showed that a bare frame is not adequate for this type of ground motion and the maximum absolute displacement reached more than 450mm which is a considerably large displacement that indicates an unseating and total damage. By utilizing a variety of AVSR models, the frame response has improved and displacement has been significantly reduced. Fig 19 illustrates the displacement, velocity and acceleration of a bare frame and a frame with models C and D respectively. The displacement decreased by about 63% to 80% for all models. A frame with the D3 model demonstrated the best performance, while a frame with the C3 model showed the lowest displacement reduction. Maximum velocity declination of around 58% resulted from equipping the frame with model D3 AVSR.

Furthermore, the absolute maximum value of velocity decreased by about 24% by adding D6 model which has the lowest velocity drop, while the other models reduced velocity by approximately 29% to 54%. The acceleration response has also been shown. Hence, using different geometry of AVSR has led to enhancement of frame acceleration response against severe turkey ground motion. The maximum absolute acceleration has decreased by roughly 25% to 64% which indicates a considerable impact of AVSR on frame acceleration responses subjected to strong seismic excitation. As a result, the overall frame response to the Turkey earthquake has been enhanced. However, displacement reduction was significantly better than velocity and acceleration response since the proposed device was a stiffness dependent system and the difference in reduction between models was due to capacity variation.

**Fig 20:**
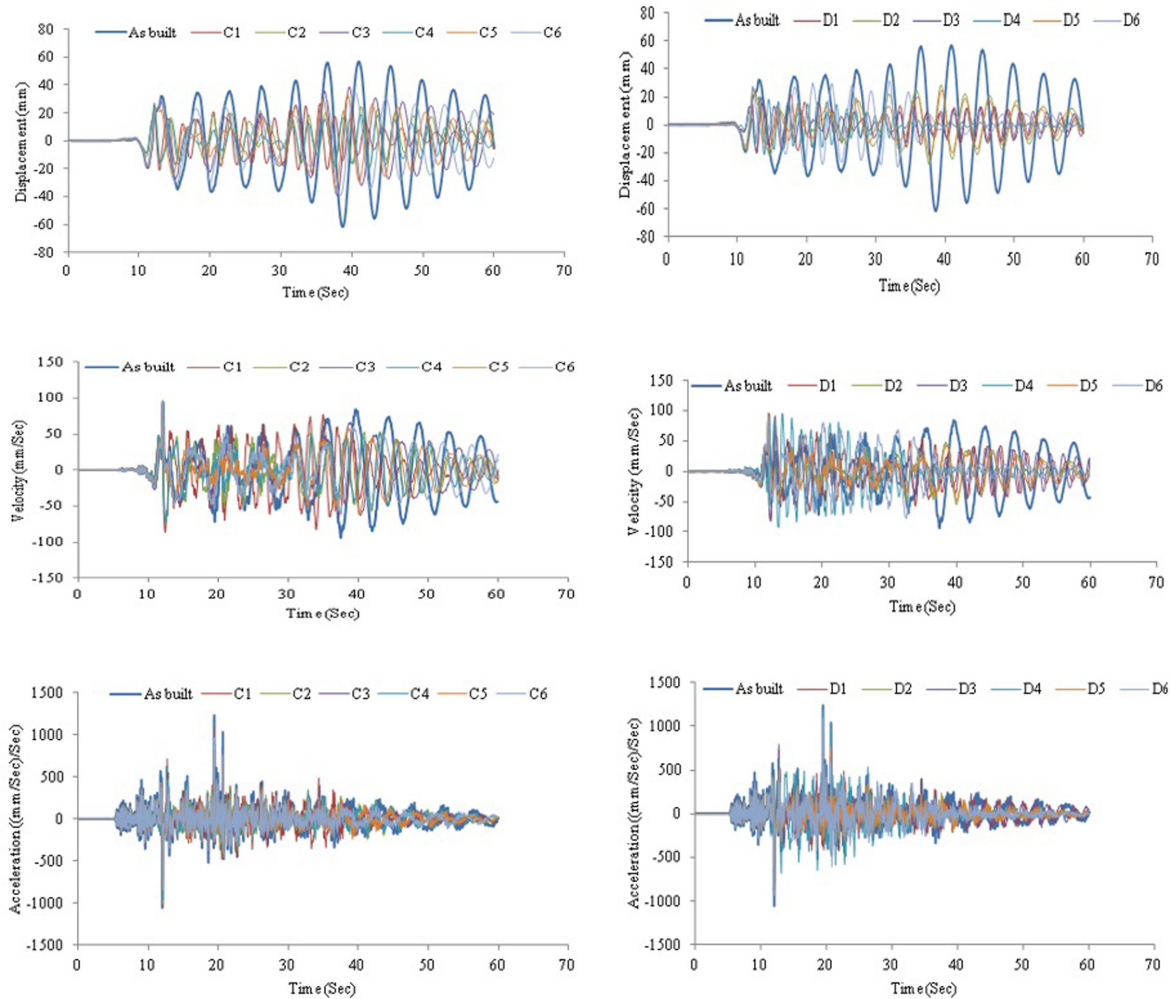
Structural responses for frame without and with C and D device models under Chi-Chi excitation.

**Fig 21:**
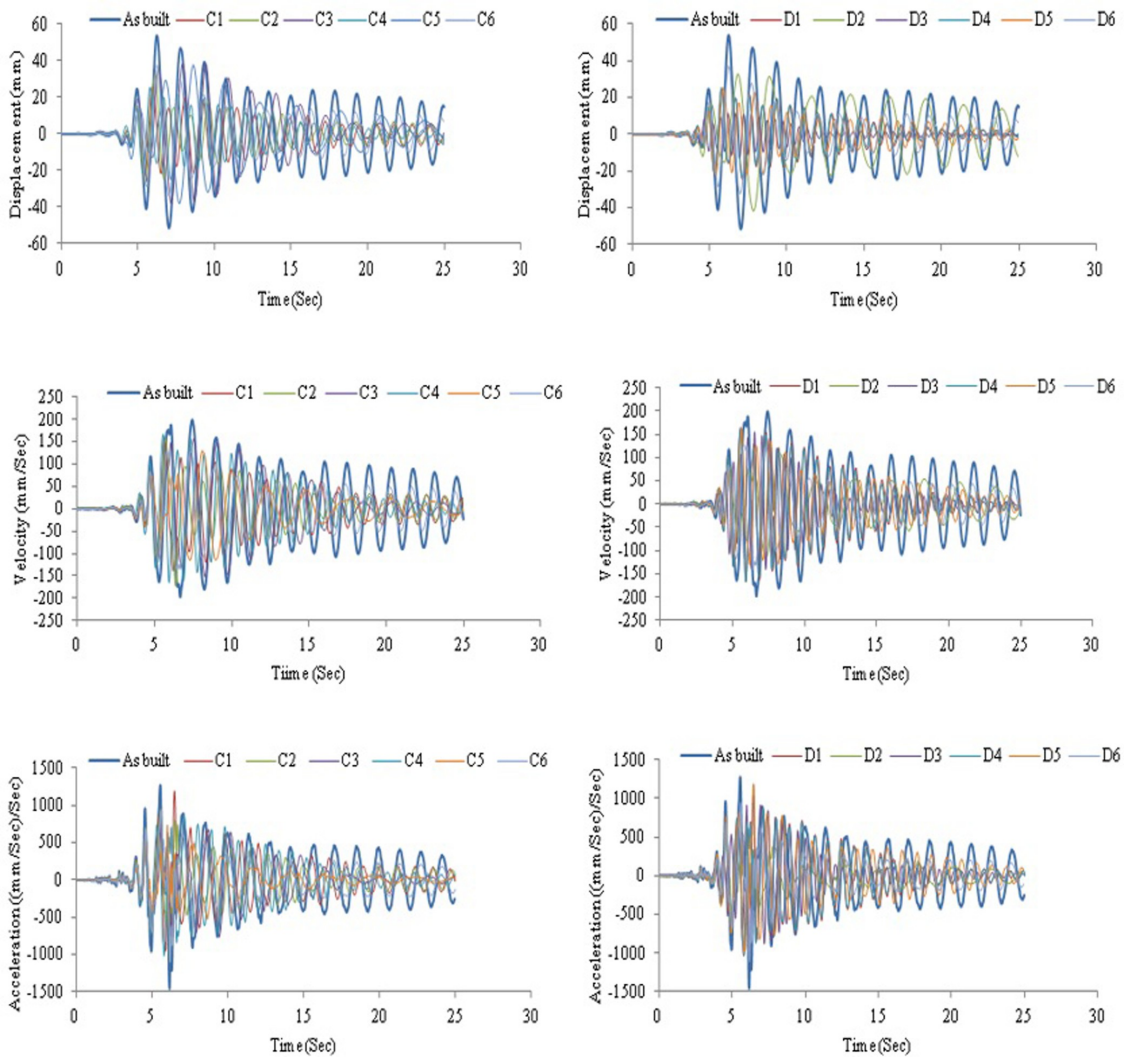
Structural responses for frame without and with C and D device models under Kobe excitation.

The bridge frame response was assessed with another two earthquake records Chi-Chi and Kobe ground motions. The outputs demonstrated a considerable impact on bare frame behavior. Though, adding AVSR has improved the frame response against the mentioned earthquakes. Fig 20 shows displacement, velocity and acceleration responses for bare frame and frame equipped C and D model AVSRs subjected to Chi-Chi excitation. Hence, frame response improvement was interpreted from significant displacement, velocity and acceleration declines after applying different configurations of AVSR. Model D3 has decreased maximum absolute displacement by roughly 66%, which represents the maximum reduction among all frame models, while other models dropped displacement between 31% and 64%. The results for velocity response against Chi-Chi ground motion revealed that frame with models C4, C2, D1, and C1 showed significant velocity diminishing of about 40%, 28%, 21% and 19% respectively. While, the frame with models D3, D5 and D6 demonstrated slight velocity dropping. On the other hand, maximum absolute velocity has increased in five models, C3, C5, C6, D2 and D4 correspondingly. In addition, the absolute maximum value of acceleration was reported to have decreased considerably in all frame models with different specifications of AVSR. Thus, frame with models D4, D6 and C1 showed declines of around 4%, 11% and 13% respectively. Nevertheless, the other models accelerations have decreased by 22% to 28%. As a consequence, AVSR verified its appropriateness for diminishing Chi-Chi ground motion. Fig 21 shows the displacement, velocity and acceleration vs. time for a bare frame and a frame with C and D type AVSR respectively subjected to Kobe earthquake. Essentially, utilizing AVSR in a bridge frame subjected to Kobe excitation causes a reduction in the maximum displacement, velocity and acceleration. Thus, the best enhancement of displacement response has been achieved by adding D3 model AVSR and 78% was the percentage of decline using D3 model while the other device configurations have decreased displacement by about 29% to 53%. Furthermore, comparison in velocity response of bare frame and implementation of different AVSR properties in frame model toward Kobe earthquake have been demonstrated as well. The absolute maximum value of velocity decreased by about 17% to 46% for all models and it is considered a significant reduction against such type of ground motion. Moreover, the maximum absolute acceleration declined by 6% to 42% compared to bare frame.

Table 5 illustrates the frame response reduction subjected to different seismic records using different configurations of AVSR. In view of the fact that the displacement, velocity, and acceleration frame responses are influenced by different characteristics of AVSR such as spring wire section, mean diameter, and number of coils. Therefore, there were differences in frame responses, and some of these parameters may improve the response by increasing their values. However, other characteristics aggravate the frame seismic response. For example, model D3 significantly enhanced all frame responses, while model C3 in general slightly improved displacement but caused acceleration incrementally during different types of earthquakes. Generally, the results revealed that the added AVSR has a significant impact on frame response and showed a considerable reduction in displacement, velocity and acceleration. Moreover, the outputs demonstrated that the AVSR able to meet the design criteria for applications of the proposed AVSR as an unseating prevention device. The results also showed that rectangular wire type AVSR has a more significant effect on frame response than circular type in all applied ground motions.

**Table 5:** Reduction in frame responses subjected to different ground motions using different configurations of AVSR.

	El-Centro			Turkey			Chi-Chi			Kobe		
Model	dis%	vel%	acc%	dis%	vel%	acc%	dis%	vel%	acc%	dis%	vel%	acc%
C1	48.9	29.9	8.4	73.5	49.5	64.0	53.5	18.6	12.8	52.1	20.0	5.9
C2	45.6	23.7	8.0	73.0	32.6	32.8	51.6	27.8	22.1	40.5	22.5	26.1
C3	18.6	12.0	-11.2	63.0	38.7	43.4	31.4	-0.9	23.1	28.8	22.0	36.1
C4	50.6	52.4	15.3	77.4	49.6	37.0	54.2	40.3	23.5	52.8	17.4	31.5
C5	24.0	3.2	-1.9	68.7	37.1	29.7	41.1	-1.5	24.3	30.2	35.8	42.4
C6	22.6	5.4	7.1	63.9	28.9	42.2	39.3	-0.4	21.8	30.0	34.9	27.0
D1	50.9	45.8	15.7	77.5	54.5	45.8	61.6	21.0	27.7	52.8	17.4	31.5
D2	31.0	12.2	-20.4	73.0	53.0	64.0	50.3	-1.1	25.3	39.3	46.1	39.9
D3	74.4	45.4	6.6	80.6	57.8	44.8	66.4	2.8	28.3	78.4	22.7	17.6
D4	73.4	43.3	7.1	77.7	42.5	36.4	64.2	-1.9	4.4	53.4	18.7	31.5
D5	49.1	54.0	16.7	77.3	46.1	47.0	53.9	0.2	25.8	52.8	18.9	6.8
D6	30.2	3.4	0.9	69.0	24.1	24.8	44.2	6.9	11.0	30.8	35.3	26.1

Note: (-) indicats incrimental in value

**Fig 22:**
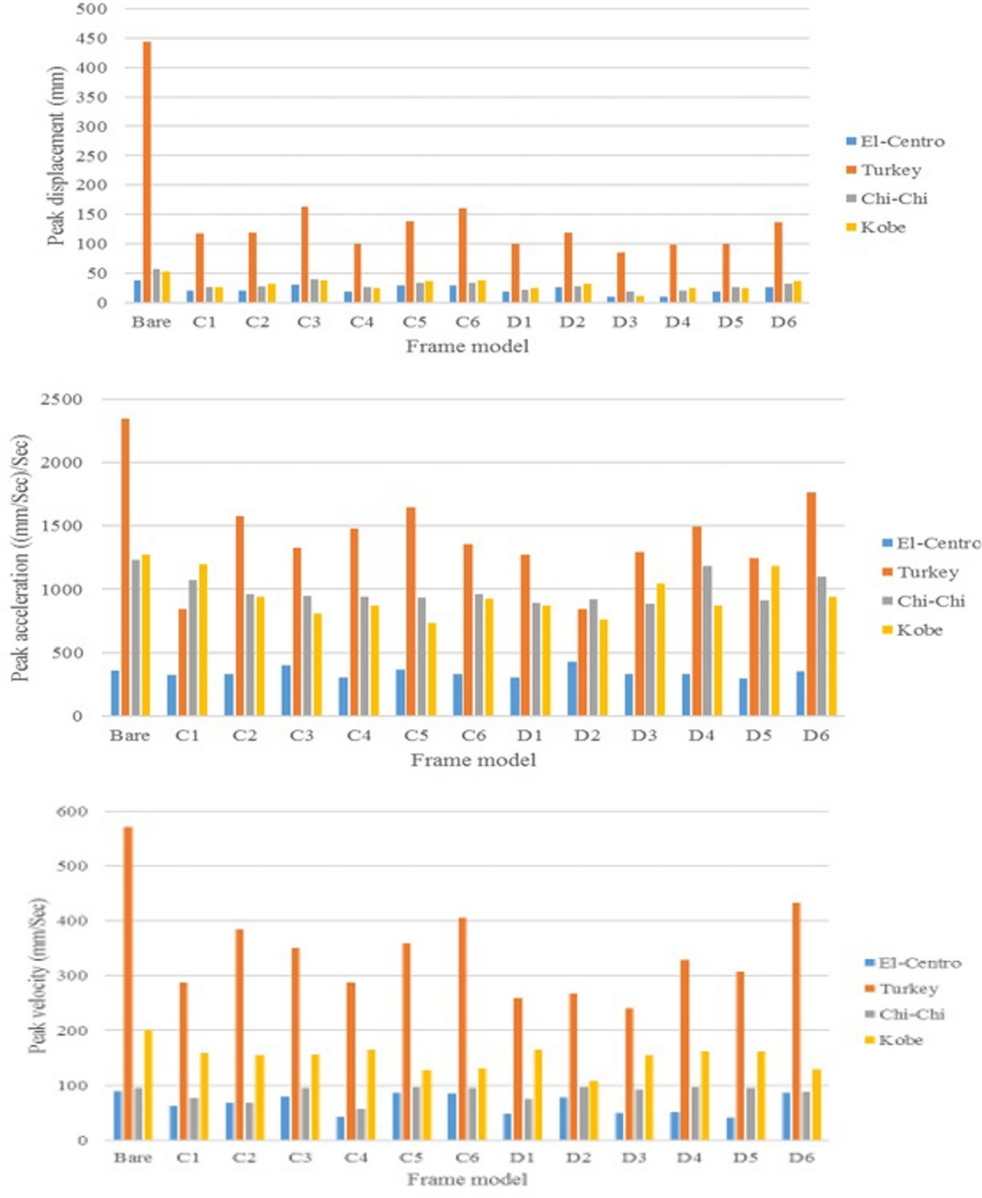
Peak responses for bare frame and frame equipped with different configurations of AVSR subjected to different earthquake records.

The effect of seismic excitation characteristics such as duration and intensity on AVSR performance was evaluated by comparing the peak responses of bare frame and frame with different device parameters. The selected earthquakes were El-Centro, Turkey, Chi-Chi and Kobe excitations had different intensities and durations. Fig 22 shows the comparison of frame structural responses between a bare frame and an equipped frame. The results revealed that displacement of a bare frame subjected to severe Turkey excitation was considerably larger than other ground motions due to long duration and large intensity. Furthermore, applying Chi-Chi ground motion to bare frame resulted in more than 50mm displacement which is slightly more than a frame subjected to Kobe earthquake while 38mm was the maximum displacement resulting from El-Centro excitation. By adding different configurations of AVSR, the displacement response was improved with all applied earthquakes. However, a frame equipped with AVSR subjected to Turkey excitation showed displacement between 85mm to 165mm which is significantly large. Therefore, the best configurations of AVSR should be selected in order to overcome the severe impact of such type of ground motion. On the other hand, the currently used device specifications are suitable for frame subjected to the remaining selected seismic excitations. The variation of seismic excitation durations and intensities also resulted in a different and considerable effect on the frame velocity response. The outputs indicated that the strong Turkey excitation has the most significant impact on frame velocity responses and the maximum absolute velocity of bare frame was more than 550mm/Sec. In addition, Kobe ground motion also had a meaningful effect on frame maximum absolute velocity at around 200mm/Sec. While the remaining two earthquakes showed less than 100mm/Sec frame peak velocity. By equipping frames with AVSRs, velocity response has improved by some device characteristics such as model D3. However, other models caused velocity increments, and frames subjected to severe Turkey excitation still showed large velocities compared with models subjected to other earthquakes. As a consequence, variation in ground motion properties will cause different impacts on frame and AVSR behavior. The last assessed response was frame acceleration against applied seismic excitations since it has an essential impact induced by vibration. As same displacement and velocity responses, the maximum acceleration response of a bare frame subjected to strong Turkey ground motion was more than 2200 mm/Sec^2^ which is almost double responses of a frame affected by Chi-Chi and Kobe earthquakes while El-Centro ground motion has caused less than 400 mm/Sec^2^.In addition, the results showed acceleration improvement by adding some types of AVSR. However, three frame models with the proposed restrainer subjected to El-Centro earthquake have shown incremental acceleration. To sum up, the efficiency of AVSR in mitigating the ground motion effect was proven to be significant using the SDoF system. In addition, earthquake properties, frame characteristics, and restrainer parameters have a significant impact on frame seismic response. Since the frame and applied earthquake will not be changed for existing bridges, AVSR should be utilized as a supplement to retrofitting restrainers, and the suitable device characteristics have to be considered in each bridge.

## Conclusions

The current research demonstrated the development of an adjustable variable stiffness restrainer (AVSR) applicable for bridges as an unseating prevention system. A small prototype of the AVSR was fabricated and tested under incremental and cyclic loading in order to assess the restrainer’s performance, and the behavior has been validated through numerical simulation. The constitutive model of AVSR has been derived, and a parametric study has been conducted numerically to evaluate the effects of different parameters on AVSR capacity. Moreover, the developed constitutive models have been implemented to conduct time history analysis for a single degree of freedom system equipped with AVSR subjected to El-Centro, Turkey, Chi-Chi and Kobe ground motions using the Newmark’s method in order to assess the dynamic behavior of AVSR. The experimental results showed a good agreement with the finite element results, and the variable stiffness behavior of the system has been observed. The parametric study results indicated that by increasing the section area of the spring wire, the restrainer performance is improved. However, the incremental changes in mean spring diameter and number of coils for each spring in AVSR resulted in a decline in the restrainer resistance. The results of the dynamic analysis of bare frames and frames equipped with different configurations of AVSR demonstrated that the frame response in terms of displacement, velocity, and acceleration was greatly improved by adding the restrainer. Furthermore, the seismic excitation properties, such as intensity, are considered important factors in the design parameters of the AVSR. The proposed device is a displacement-based restrainer that has no ability to dissipate energy. Thus, the restrainer is able to prevent the bridge from unseating due to an earthquake. In the future, some modifications will be required to improve the device’s performance in terms of energy dissipation possibilities, such as adding a dashpot or rubber material.
